# Investigation of Flavonoid Scaffolds as DAX1 Inhibitors against Ewing Sarcoma through Pharmacoinformatic and Dynamic Simulation Studies

**DOI:** 10.3390/ijms24119332

**Published:** 2023-05-26

**Authors:** Muhammad Yasir, Jinyoung Park, Eun-Taek Han, Won Sun Park, Jin-Hee Han, Yong-Soo Kwon, Hee-Jae Lee, Mubashir Hassan, Andrzej Kloczkowski, Wanjoo Chun

**Affiliations:** 1 Department of Pharmacology, Kangwon National University School of Medicine, Chuncheon 24341, Republic of Korea; yasir.khokhar1999@gmail.com (M.Y.); jinyoung0326@kangwon.ac.kr (J.P.); heejaelee@kangwon.ac.kr (H.-J.L.); 2 Department of Medical Environmental Biology and Tropical Medicine, Kangwon National University School of Medicine, Chuncheon 24341, Republic of Korea; ethan@kangwon.ac.kr (E.-T.H.); han.han@kangwon.ac.kr (J.-H.H.); 3 Department of Physiology, Kangwon National University School of Medicine, Chuncheon 24341, Republic of Korea; parkws@kangwon.ac.kr; 4 College of Pharmacy, Kangwon National University School of Medicine, Chuncheon 24341, Republic of Korea; yskwon@kangwon.ac.kr; 5 The Steve and Cindy Rasmussen Institute for Genomic Medicine at Nationwide Children’s Hospital, Columbus, OH 43205, USA; mubashirhassan_gcul@yahoo.com (M.H.); andrzej.kloczkowski@nationwidechildrens.org (A.K.)

**Keywords:** Ewing sarcoma, DAX1, flavonoids, molecular docking, pharmacogenomics, molecular dynamics simulation

## Abstract

Dosage-sensitive sex reversal, adrenal hypoplasia critical region, on chromosome X, gene 1 (DAX1) is an orphan nuclear receptor encoded by the *NR0B1* gene. The functional study showed that DAX1 is a physiologically significant target for EWS/FLI1-mediated oncogenesis, particularly Ewing Sarcoma (ES). In this study, a three-dimensional DAX1 structure was modeled by employing a homology modeling approach. Furthermore, the network analysis of genes involved in Ewing Sarcoma was also carried out to evaluate the association of DAX1 and other genes with ES. Moreover, a molecular docking study was carried out to check the binding profile of screened flavonoid compounds against DAX1. Therefore, 132 flavonoids were docked in the predicted active binding pocket of DAX1. Moreover, the pharmacogenomics analysis was performed for the top ten docked compounds to evaluate the ES-related gene clusters. As a result, the five best flavonoid-docked complexes were selected and further evaluated by Molecular Dynamics (MD) simulation studies at 100 ns. The MD simulation trajectories were evaluated by generating RMSD, hydrogen bond plot analysis, and interaction energy graphs. Our results demonstrate that flavonoids showed interactive profiles in the active region of DAX1 and can be used as potential therapeutic agents against DAX1-mediated augmentation of ES after in-vitro and in-vivo evaluations.

## 1. Introduction

Ewing sarcoma family tumors are distinguished by the presence of non-random chromosomal translocations that result in fusion genes that encode aberrant transcription factors. EWS RNA binding protein 1 (EWSR1)-FLI-1 (Friend leukemia integration 1) emergence is caused by the t(11;22) (q24;q12) translocation, which is connected to 85% of malignancies [1]. The remaining 10% to 15% of cases are caused by the t(21;12) (22;12) translocations, which result in EWSR1-ERG (ETS transcription factor) fusion [2]. This fusion protein drives the expression of genes involved in cell proliferation and survival, leading to the development of Ewing sarcoma [1,2].

DAX1, also known as *NR0B1*, is a transcriptional regulator that plays a critical role in the development and function of several endocrine glands, including the adrenal gland and the hypothalamus [3]. Recent studies have also implicated DAX1 in the pathogenesis of Ewing sarcoma (ES), a rare type of bone cancer that primarily affects children and young adults. DAX1 is overexpressed in Ewing sarcoma and interacts with the EWS-FLI1 fusion protein [4,4,5]. DAX1 has also been shown to enhance the transcriptional activity of EWS-FLI1 by recruiting co-activators to the fusion protein. This enhanced activity promotes cell proliferation and inhibits apoptosis, contributing to the development and progression of ES [6]. It has been demonstrated that DAX1 (*NR0B1*), an orphan nuclear receptor, is induced by the EWS/FLI1 oncoprotein and is highly expressed in Ewing’s tumors, suggesting that DAX1 is a biologically relevant target of EWS/FLI1-mediated oncogenesis [4]. DAX1 is a direct transcriptional target of the EWS/FLI1 oncoprotein through its binding to a GGAA-rich region in the DAX1 promoter and shows that DAX1 is a key player in EWS/FLI1-mediated oncogenesis [7]. DAX1 silencing using an inducible model of RNA interference induces growth arrest in the A673 Ewing’s cell line and severely impairs its capability to grow in semisolid medium and form tumors in immunodeficient mice [4,8]. Gene expression profile analysis demonstrated that about 10% of the genes regulated by EWS/FLI1 in Ewing’s cells are DAX1 targets, confirming the importance of DAX1 in Ewing’s oncogenesis [9]. Functional genomic analysis, validated by quantitative RT–PCR, showed that genes implicated in cell-cycle progressions, such as CDK2, CDC6, MCM10, and SKP2 were similarly regulated by EWS/FLI1 and DAX1 [4]. These findings indicate that DAX1 is important in the pathogenesis of Ewing’s family of tumors, identifying new functions for DAX1 as a cell-cycle progression regulator and opening the possibility to new therapeutic approaches based on DAX1 function interference. In addition to its role in Ewing sarcoma pathogenesis, DAX1 may also be a potential therapeutic target for the treatment of this disease. Inhibition of DAX1 expression or function has been shown to reduce the growth and survival of Ewing sarcoma cells in vitro and in vivo [5,10].

In this study, the in-silico inhibition of DAX1 was carried out utilizing flavonoid compounds. The DAX1 3D stricture was not available on PDB therefore the 3D structure prediction of DAX1 was accomplished by homology modeling and the quality prediction of the DAX1 model was evaluated by ProSA webserver and 100 ns MD simulation. Gene network analysis was also carried out to predict the involvement of DAX1 and other genes in ES. Furthermore, the commercially available flavonoid compounds were accessed and screened by Lipinski’s Rule of 5 (RO5) and 2D and 3D structural analysis. Furthermore, the molecular docking approach was executed for DAX1 inhibition and the top ten docked flavonoid compounds were subjected to pharmacogenomics analysis to find the possible drug-to-gene interaction against ES. Therefore, the top five compounds luteolin, quercetin, kaempferol, flavopiridol, epigallocatechin, and ifosfamide as reference compounds were indulged to 100 ns MD simulation and analyzed by RMSD, hydrogen bond plot analysis, MD interaction energy analysis, and MD binding mode analysis. Our study showed promising results against DAX1 (ES) and luteolin, kaempferol, and epigallocatechin remain sustained in the active region of DAX1 and blocked its active binding pocket.

## 2. Results

### 2.1. Structural Prediction and Validation of the DAX1 Protein

**Figure 1:**
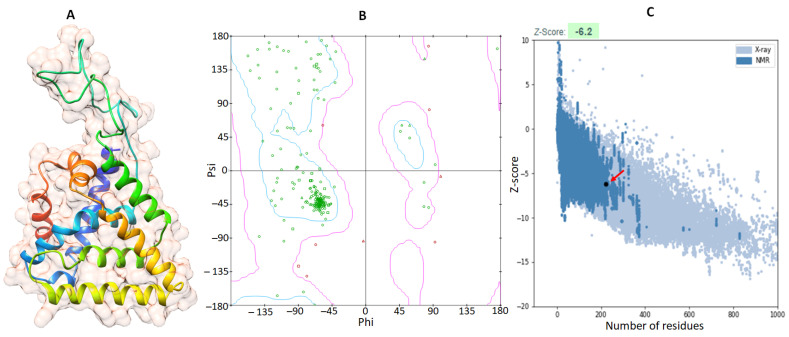
The 3D structure of the DAX1 protein is on the left side mentioned as (**A**), while the computed Ramachandran plot is on the right side mentioned as (**B**). The amino acid residues are colored as green while the favorable region is mentioned by blue boundaries and disallowed region is highlighted by pink color. Therefore, ProSA graphical depiction is exhibited in (**C**) in which the comparison of predicted protein with PDB X-ray structures is colored light blue while the comparison to NMR structures is depicted in blue.

The 3D structure of DAX1 was predicted using *Mus musculus* as a reference structure (PDBID-3F5C) with a sequence similarity of 67.16%. The 3D structure was predicted for the amino acid range of 250–470 (Supplementary Materials Figure S1). Loops, α-helices, and β-sheets occur in the overall predicted protein’s structure. The VADAR 1.8 structural results revealed that the predicted structure is made up of 57% α-helices, 1% β-sheets, 40% coils, and 21% turns, respectively. Furthermore, Ramachandran plots showed that 81.0% of amino acids occur in the favored region while 94.2% of residues were present in the allowed zone of dihedral angles phi (φ) and psi (ψ) (Figure 1A,B). Moreover, the ProSA structural evaluation of the DAX1 model reveals Z-score = −6.5. The negative value of the Z-score exhibits a good structural comparison of predicted protein with already available X-ray and NMR structures on PDB.

**Figure 2:**
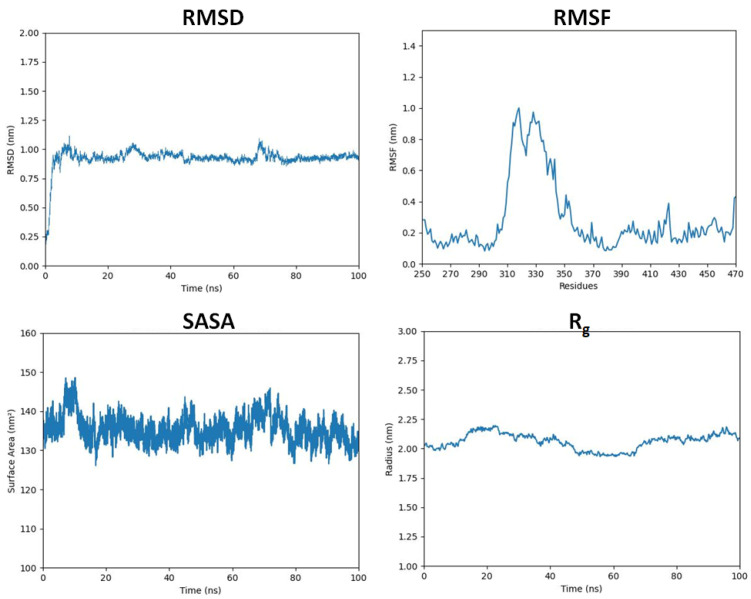
The graph shows the RMSD, RMSF, SASA, and radius of gyration of the modeled protein structure.

The RMSD analysis of DAX1 shows that predicted proteins have stable RMSD values a little fluctuation can be seen at 22 ns and 70 ns where the bar line increases the RMSD values but then again returned to its original position. The RMSF results in DAX1 show that all residues dynamically fluctuated from N to C terminals. The protein structures are composed of 250–470 AA with different structural architectures. A couple of peaks have been observed at both terminal regions. The remaining parts of all protein structures in Figure 2 remained stable throughout the simulation time (0–100 ns). Moreover, the central loop (310–360 AA) region of the protein can be seen more fluctuating due to non-secondary confirmation (Figure 2). However, these variations do not cause much disturbance in the protein conformations, which ensures that our predicted results are much more stable and steadier in behavior.

The structural compactness of the protein was calculated by the radius of gyration (R_g_). The generated results depicted that the R_g_ values of the predicted protein model showed no bigger fluctuation and from 0 ns to 15 ns the bar lines showed an increasing pattern and then it started decreasing till 50 ns and the bar line remain unfluctuating till 70 ns and then the bar line starts increasing. However, no bigger fluctuation was seen and the R_g_ remain between 2.00–2.24 till the 100 ns MD simulation. The solvent-accessible surface areas (SASAs) were also observed and are shown in Figure 2. The results showed that the values of SASA of the predicted protein model were centered between 130–140 nm^2^ mostly throughout 100 ns MD simulation.

### 2.2. Gene Network Analysis of DAX1 (NR0B1)

**Figure 3:**
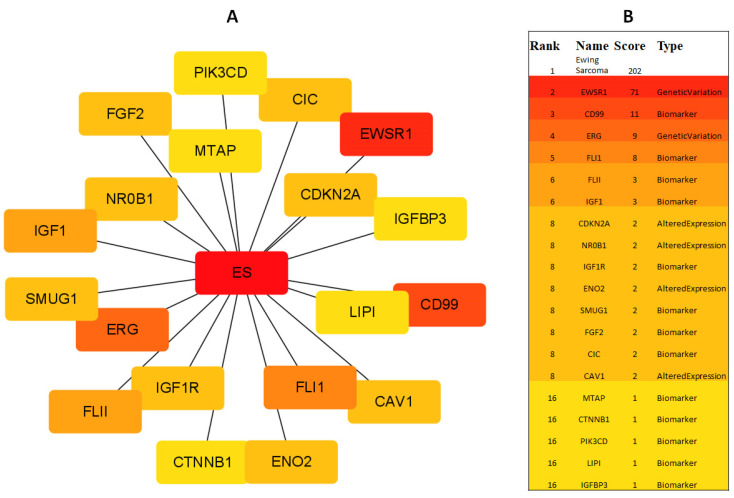
The figure demonstrates the interacting protein to ES (**A**) and the type of interaction (**B**). The DAX1 is represented as *NR0B1* at the 8th rank.

The network analysis of DAX1 (Figure 3A,B) revealed that more than two hundred genes have been linked with ES through direct or indirect expression patterns. Through which the top 20 were sorted using the gene rank approach the full-length table provided in Supplementary Materials Table S1. Therefore, the graphical depiction of the top 20 genes was carried out based on their ranks and involvement type.

### 2.3. The Binding Pocket Analysis

**Figure 4:**
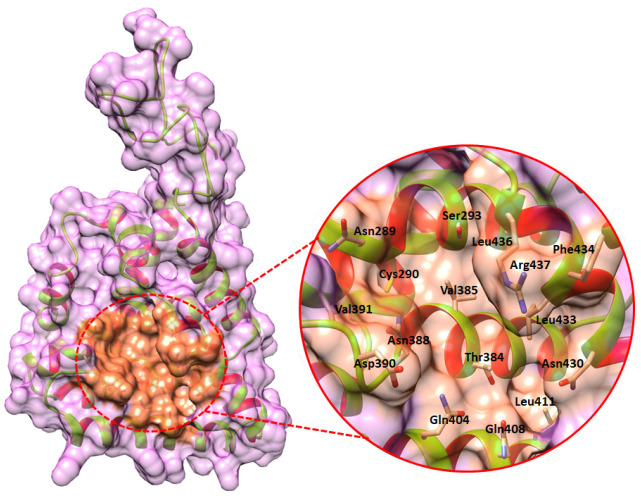
The binding pocket is positioned in coral color while the whole protein surface is colored as an orchid. Furthermore, the ribbon and helix interiors are colored chartreuse and red respectively.

A binding pocket’s function is determined by the collection of amino acid residues that surround it, in addition to its shape and location inside a protein [11]. PrankWeb is an online resource providing an interface to P2Rank, an inventive method for ligand binding site prediction. In generated results, six predicted binding pockets have been observed and ranked according to scoring values to obtain the best binding site of DAX1 (Table 1). The top ranked binding pocket residues were Asn289, Cys290, Ser293, Thr384, Val385, Asn388, Asp390, Val391, Gln404, Gln408, Leu411, Asn430, Leu433, Phe434, Leu436, Arg437, respectively (Figure 4).

### 2.4. Flavonoid’s Cheminformatics Analysis

**Figure 5:**
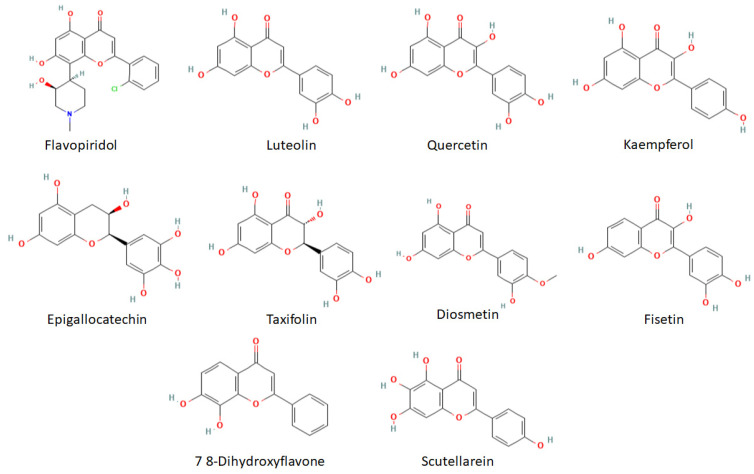
The 2D structural presentations of the top 10 screened flavonoids.

Flavonoids have several favorable biochemical, anti-cancer, anti-oxidant, anti-allergic, and anti-inflammatory characteristics against multiple diseases such as carcinogenesis, osteosarcoma, and lung cancer [12,13,14,15,16]. Based on the prior analysis, 132 flavonoid compounds were selected in SDF format. The cheminformatics analysis (molecular weight, hydrogen bonds donor, hydrogen bonds acceptors, LogP, and the number of rotatable bonds) was carried out for 132 flavonoid compounds (Supplementary Materials Table S3). Therefore, out of 132, 10 are depicted in the 2D representation (Figure 5).

### 2.5. Molecular Docking Analysis

All flavonoids-DAX1 docking results showed good scoring values and the best docked complexes were screened out based on the minimum docking energy values and binding interaction (Table 2). The ten corresponding ligands had good binding energy values and were bound to the target protein’s active region. All the rest compounds docking energies are predicted in Supplementary Materials in Table S2.

CDocker Energy, which comprises the internal energy of the ligand and the ligand’s de-solvation energy, is the overall energy score determined for the protein–ligand complex in certain docking poses. It indicates the protein–ligand complex’s overall stability and is used to rank various docking spots. On the other side, CDocker Interaction Energy is the energy score that is determined just for the protein–ligand interaction, eliminating the internal and de-solvation energies of the ligand. It reflects the potency of certain interactions, such as hydrogen bonds, van der Waals interactions, and electrostatic interactions, between the protein and the ligand.

### 2.6. Pharmacogenomics Analysis

The top ten flavonoids with significantly low docking energy were further investigated using pharmacogenomics analysis. Pharmacogenomics aims to be a realistic way to optimize drug therapy in relation to the genotype of the patient to achieve maximum efficacy with a minimum of negative effects [17]. The predicted genes for flavonoids have been evaluated based on interaction score values. The comparative results showed that luteolin exhibited a good interacting profile with the TNFRSF10B gene with an interaction score of 0.55 which have a direct association with ES. Another drug, quercetin also showed good association with ES by interacting CD34 gene having an interaction score of 0.39. Flavopiridol was also discovered to target ES by interacting AR gene (Table 3).

### 2.7. Binding Interaction Analysis

Binding analysis was carried out for the top five flavonoid compounds which exhibit the lowest molecular docking energy and good pharmacogenomic association against ES. Luteolin, quercetin, and flavopiridol were targeting the genes which were involved in ES and the rest are targeting bone sarcoma, childhood osteosarcoma, osteosarcoma of bone, and others.

**Figure 6:**
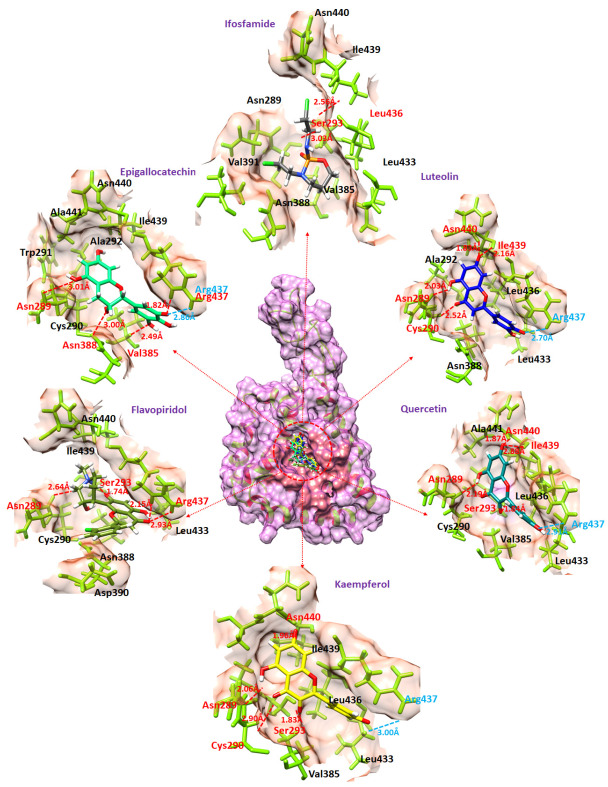
This figure shows the binding of flavonoid compounds in the active region of the DAX1 protein. The amino acids which are making hydrogen bonds and the binding distances are colored red while the amino acids which are producing salt bridges and the bonding are colored cyan. Furthermore, each flavonoid compound is colored differently (Luteolin (blue), Quercetin (dark cyan), Kaempferol (yellow), Flavopiridol (olive), Epigallocatechin (spring green), and Iforfamide (dim grey).

Luteolin compound manifests the lowest molecular docking energy in molecular docking analysis. The luteolin-DAX1 complex exhibited that the ligand formed four hydrogen bonds and one salt bridge against Asn440, Ile439, Asn289, Cys290, and Arg437 with bonding distances of 1.83 Å, 2.16 Å, 2.03 Å, 2.52 Å, and 2.70 Å respectively (Figure 6). The quercetin–DAX1 docked complex also exhibited four hydrogen bonds and one salt bridge against Asn440, Ile439, Asn289, Ser293, and Arg437 with bonding distances of 1.87 Å, 2.88 Å, 2.19 Å, 1.84 Å, and 2.91 Å. Furthermore, the kaempferol compound, docked to DAX1, formed four hydrogen bonds and a salt bridge against Asn440, Asn289, Cys290, Ser293, and Arg437 with bonding lengths of 1.96 Å, 2.06 Å, 2.90 Å, 1.83 Å, and 3.00 Å, respectively. The Flavopiridol docked complex predicts four hydrogen bonds against Asn289, Ser293, and Arg437 with a bond length of 2.64 Å, 1.74 Å, 2.15 Å, and 2.93 Å. The same oxygen atom made two hydrogen bonds with Arg437 with different bonding distances. Moreover, the receptor–ligand complex of epigallocatechin and DAX1 depicts four hydrogen bonds and a salt bridge against Asn289, Asn388, Val385, and Arg437 with bonding lengths of 3.01 Å, 3.00 Å, 2.49 Å, 1.82 Å, and 2.86 Å. The oxygen atom of epigallocatechin formed one hydrogen bond and one salt bridge against Arg437. Comparatively, the ifosfamide drug docked to receptor (DAX1) predicts two hydrogen bonds against Leu436 and Ser293 with bonding distances of 2.56 Å and 3.03 Å respectively.

### 2.8. Molecular Dynamics Simulation

The top five compounds which exhibited the lowest molecular docking energies and good pharmacogenomics results were subjected to MD simulation. The 100 ns MD simulation was carried out for each complex and the stability of docked complexes was evaluated in root mean square deviation (RMSD) hydrogen bond Plot analysis, MD interaction energy analysis, and MD binding analysis. Furthermore, the receptor was also subjected to MD for receptor RMSD, RMSF (root mean square fluctuation), Radius of gyration (Rg), and SASA (solvent accessible surface area) calculation and analysis.

### 2.9. RMSD Analysis

Using GROMACS, 100 ns long MD simulations were run to assess the flexibility and general stability of the docked complexes. Using RMSD from MD trajectories, the variations of ligands inside the DAX1 protein’s active region were identified.

**Figure 7:**
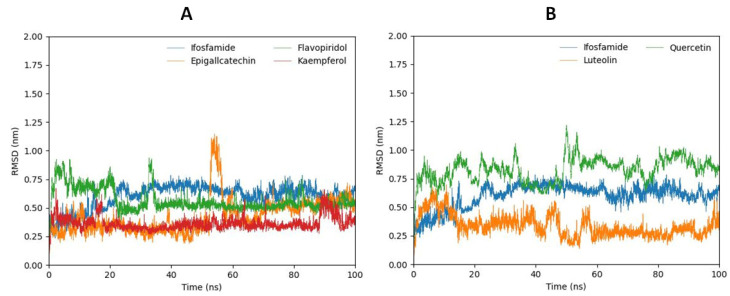
The bar graphs (**B**) represent the RMSD analysis of luteolin (orange), and quercetin (green) in comparison with ifosfamide (blue). Graph (**A**) represents the RMSD of kaempferol (red), flavopiridol (green), and epigallocatechin (orange) in comparison with ifosfamide (blue).

The luteolin compound, which manifests the lowest molecular docking energy, predicts relatively stable RMS deviations during 100 ns MD simulation. At the start, the bar line increased the RMSD, and then after 18 ns, the bar line showed a stabilizing pattern. Furthermore, the quercetin compound, which depicts lower docking energy (−45.4411) following luteolin, showed a highly fluctuating bar line and high RMSD values. Moreover, the kaempferol compound exhibited the most stable RMSD. The bar line fluctuated only once from 85 ns to 90 ns and increased its RMS devotion to σ = 0.50. The flavopiridol compound also maintained stable RMSD from 38 ns to 100 ns. The bar line increased RMSD at the start and then suddenly dropped to σ = 0.50 at 22 ns. The epigallocatechin compound also depicts the stable bar line and low RMSD values; therefore, after reaching 52 ns, the bar line dramatically fluctuates and increases the RMS deviation, and from 65 ns to 100 ns the bar line changes its position to σ = 0.50. Furthermore, ifosfamide was also carried out through RMSD analysis for comparative study (Figure 7A,B). The ifosfamide RMSD analysis reveals that the bar increases the RMSD values at the start and then after reaching 30 ns the RMSD values of ifosfamide remained stable at σ = 0.60 until 100 ns.

### 2.10. Hydrogen Bond Plot Analysis

In the hydrogen bond plot analysis, types of hydrogen bonds were distinguished based on their bonding distance: those with a bonding distance of less than 0.35 Å and those with a length larger than 0.35 Å. Hydrogen bonds with a bond length of 0.35 Å are stronger than other hydrogen bonds because of their small bonding distance. Since the bonding lengths of other hydrogen bonds are longer than 0.35 nm, they are thought to be weaker.

**Figure 8:**
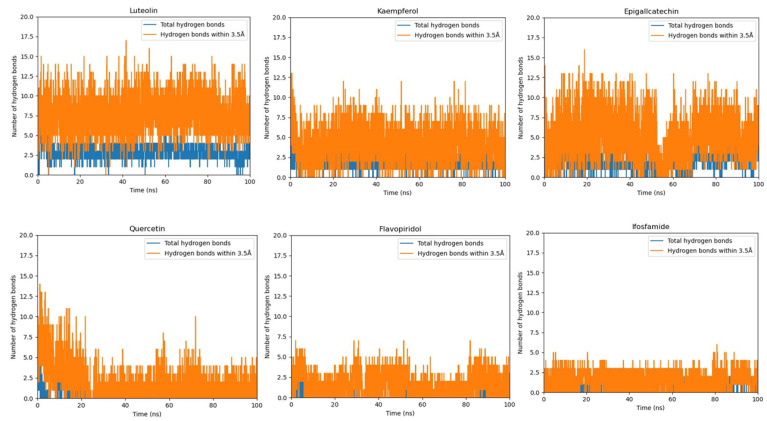
The graphs exhibit the hydrogen bond formation throughout the 100 ns MD simulation. The hydrogen bonds which are less than 0.35 Å in length are colored orange while the hydrogen bonds with bonding distances higher than 0.35 Å are colored blue.

The luteolin compound shows the highest number of hydrogen bonds under 0.35 Å. The graphical representation of luteolin shows that more than 17 hydrogen bonds were created during the 100 ns MD simulation from which five hydrogen bonds remain active throughout the 100 ns MD simulation. The quercetin compound manifests that more than twelve hydrogen bonds were created during MD simulation from which four mostly remained stable and active till 100 ns. Furthermore, the kaempferol compound, which exhibits the lowest RMSD, showed the formation of more than twelve hydrogen bonds from which five remained continuously active during the 100 ns MD simulation. Moreover, the hydrogen plot analysis of flavopiridol reveals that seven hydrogen bonds were created during MD simulation and three were most active. The epigallocatechin compound also manifested a high number of hydrogen bonds under 0.35 Å in hydrogen bond plot analysis (Figure 8). Therefore, the ifosfamide drug demonstrates that more than five hydrogen bonds were formed during MD simulation and from which three mostly remained active till 100 ns.

### 2.11. MD Interaction Energy Analysis

**Figure 9:**
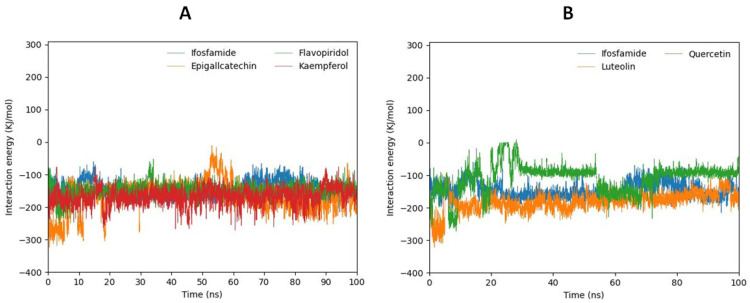
Graph (**A**) shows the MD interaction energy of flavopiridol, kaempferol, and epigallocatechin in comparison with ifosfamide. Graph (**B**) shows the MD interaction energy of Luteolin and quercetin in comparison with ifosfamide.

The interaction energy calculation was also calculated from MD trajectories. The interaction energy was calculated in two forms: electrostatic (Coulombic) interaction energy and Lennard–Jones interaction energy, with their sum representing the total interaction energy. According to interaction energy analysis luteolin, kaempferol, and epigallocatechin exhibited the lowest MD interaction energy (Table 4). Quercetin, which shows high fluctuations in RMSD and a low number of hydrogen bonds, manifested the highest MD interaction energy. Therefore, ifosfamide predicts (−147.7736) high MD interaction energy than luteolin, kaempferol, flavopiridol, and epigallocatechin. Furthermore, the interaction energy of those five compounds and reference drug is also depicted in a graphical representation (Figure 9A,B).

### 2.12. MD Binding Mode Analysis

**Figure 10:**
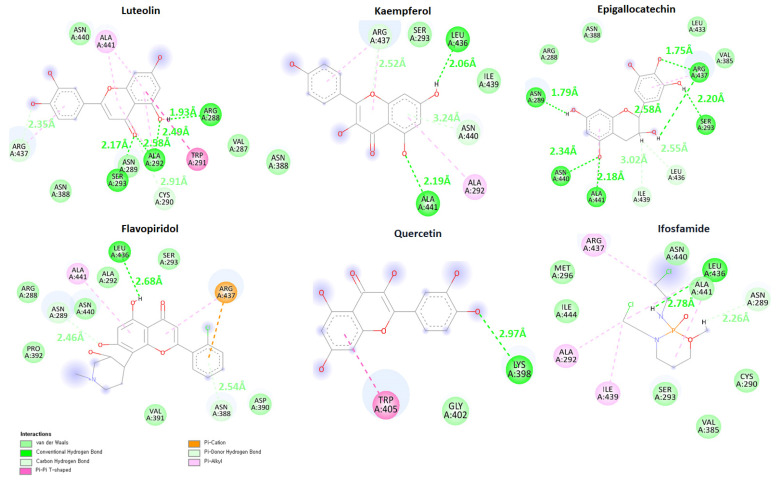
Shows the graphical depiction of binding mode analysis. The predicted conventional hydrogen bonds are colored green while carbon-hydrogen bonds are colored light green. Furthermore, the bonding distances are mentioned with each interaction and colored respectively.

To analyze the interaction of flavonoid compounds during 100 ns MD simulation MD binding mode analysis was carried out. Snapshots of all five simulated compounds and reference compound ifosfamide were retrieved at 100 ns and visualized by discovery studio and UCSF Chimera. The results demonstrate that all compounds remained in the active region of DAX1 during the 100 ns Md simulation and maintained conventional and carbon–hydrogen bonds with binding pocket amino acid residues of DAX1 except quercetin. Quercetin compound which showed a highly fluctuating RMSD graph and higher interaction energy in MD interaction energy analysis showed the lowest interaction at 100 ns. However, the luteolin, kaempferol, and epigallocatechin compounds which showed the lowest interaction energy and stable RMSD showed higher interaction and maintained conventional and carbon–hydrogen bonds until 100 ns (Figure 10). Therefore, the ifosfamide and flavopiridol compounds exhibited fewer hydrogen bonds in hydrogen bond plot analysis and also depicts fewer interactions at 100 ns in MD binding mode analysis.

## 3. Discussion

DAX1 has been identified as a potential therapeutic target for Ewing sarcoma, and several studies have investigated the use of DAX1 inhibitors to block its activity and reduce tumor growth. In preclinical studies, DAX1 inhibition has been shown to induce cell death in Ewing sarcoma cells and reduce tumor growth in mouse models [4,4,9]. This study aims to develop a potent drug against DAX1. Therefore, more than 300 commercially available flavonoid compounds were retrieved from online sources and their molecular descriptor values were predicted for the screening process. The compounds violating Lipinski rules of five (Molecular weight, Hydrogen bond donor, hydrogen bond acceptor, and LogP) were excluded from the data set at an earlier stage and energy minimization was carried out for the selected 132 compounds. Flavonoids have a common flavone backbone, which comprises two phenyl rings (A and B) and a heterocyclic ring (C). The variations in the structures of different flavonoids come from the number and arrangement of hydroxyl groups, the saturation level of the C ring, and the connection between the B ring and the C ring. On the other hand, the receptor’s 3D structure was not available on PDB therefore 3D structure was predicted using homology modeling concerning *Mus musculus* DAX1 3D structure which was the only available structure at that time. Furthermore, the DAX1 predicted structure was evaluated from online web-server VADAR, ProSA, and Ramachandran analysis was carried out for the predicted protein model. Ramachandran analysis reveals that 81.0% of amino acids occur in the favored region while 94.2% of residues were in the allowed region. ProSA exhibited a Z-score of −6.5 in comparison to already available X-ray and NMR structures on PDB and VADAR calculate the α-helix, β-sheets, and turns in the whole DAX1 model. The predicted DAX1 protein’s 3D model was also evaluated by RMSD, RMSF, SASA, and Rg (radius of gyration) in separate 100 ns MD simulations. The RMSD of DAX1 showed quite stable behavior and the bar line exhibited stable RMSD values at σ = 0.90. SASA and Rg graphs also depicted stable graphical bar lines. Therefore, the RMSF was shown to have highly fluctuating residues (300–360) due to the loop region in the central domain of the predicted DAX1 model. Moreover, the DAX1 gene network analysis was carried out to predict the possible involvement of DAX1 and other relevant genes in ES. Therefore, the top 20 genes were sorted from the data set of more than 200 genes.

So, the prepared flavonoid compounds were docked against the predicted DAX1 model separately. Moreover, the ifosfamide drug which is already a known drug against ES was also subjected to molecular docking for comparative study. Ifosfamide’s active metabolites have the ability to attach to DNA molecules and create covalent bonds with nucleotides, which can result in DNA damage and prevent DNA replication and cell division. Additionally, ifosfamide has the ability to create crosslinks between DNA strands, which can result in DNA strand breaks and lessen the ability of DNA to replicate and divide.

The results indicate better docking score values of flavonoids in comparison to ifosfamide against DAX1. Furthermore, the pharmacogenomics analysis was carried out for the top 10 docked compounds to evaluate the possible associations of those flavonoid compounds with the genes involved in ES. As a result, three of the top five flavonoid compounds (luteolin, quercetin, and flavopiridol) were targeting different genes which are directly involved in ES. Therefore, the binding interaction analysis was carried out for the top five compounds which exhibited the lowest docking score values and good associations in pharmacogenomic analysis and the results revealed the hydrogen bond, hydrophobic interactions, and salt bridges of the top five compounds in the active region of the target protein which exhibits strong correlations of flavonoid compounds against DAX1.

The top five compounds were subjected to 100 ns MD simulation and further evaluated in RMSD, Hydrogen Bond plot analysis, and MD interaction energy analysis. The quercetin compound showed high RMSD values and a low number of hydrogen bonds in the hydrogen bond plot analysis. Therefore, predicting high interaction energy in MD interaction energy analysis except that the luteolin, kaempferol, flavopiridol, and epigallocatechin showed quite stable RMSD values as compared to ifosfamide and a higher number of hydrogen bonds were predicted by the luteolin, kaempferol, and epigallocatechin in hydrogen bond plot analysis while ifosfamide showed 2.5 hydrogen bonds in hydrogen bond plot analysis. Furthermore, the MD interaction energy analysis profile of the luteolin, kaempferol, and epigallocatechin also predicted the lowest interaction energy (−181.8286, −167.573, −165.6815) as compared to ifosfamide (−147.7736). Moreover, the MD binding mode analysis revealed that all the compounds remain bounded in the active region of target DAX1 and depict good interaction. The compound ifosfamide manifests less hydrogen bond formation in hydrogen bond plot analysis and depicts the short number of interactions at 100 ns binding mode analysis. The quercetin compound, which exhibited higher RMSD values, fewer hydrogen bonds, and high interaction energy, exhibited less interaction. At the start of the simulation, quercetin depicted a high number of hydrogen bonds but after some time RMS deviation started fluctuating highly and a number of hydrogen bond formations dropped, which may the due to the excretion of the quercetin molecule from the active region of the target protein. Therefore, in binding mode analysis, it was observed that the quercetin molecule comes out from the active pocket of DAX1.

Our results showed promising results of flavonoid compounds against the predicted DAX1 model and block the active site by hindering the active site amino acid residues. Therefore, experimental or in-vitro confirmation is necessary to evaluate the enzymatic activity and further confirm enzyme inhibition.

## 4. Materials and Methods

### 4.1. DAX1 Structure Prediction and Validation

The complete structure of the DAX1 protein was not available on PDB. Therefore, the FASTA sequence was retrieved from the UniProt (ID: P51843, accessed on 2 March 2023). Furthermore, the three-dimensional (3D) structure prediction was carried out using SWISS-MODEL a fully automated protein structure homology-modeling server, accessible via the Expasy web server (https://swissmodel.expasy.org/ (accessed on 3 March 2023)), based on homology modeling with the template *Mus musculus* (PDBID-3F5C) by the sequence similarity of 67.16%. Therefore, the target protein’s energy minimization was carried out using Discovery Studio [62]. Moreover, the Ramachandran graph of DAX1 was assessed by Discovery Studio [63]. The protein architecture and statistical percentage values of helices, beta-sheets, coils, and turns were accessed by using the online tool VADAR 1.8 (http://vadar.wishartlab.com/ (accessed on 3 March 2023)).

Furthermore, the predicted model of the DAX1 protein was also analyzed by the ProSA webserver (https://prosa.services.came.sbg.ac.at/prosa.php (accessed on 10 May 2023)). The ProSA is a system for evaluating the quality of protein 3D structures that compares the protein’s overall model quality score to experimentally solved protein structures contained in the PDB database using their X-ray and NMR models. Therefore, the DAX1 model was further subjected to 100 ns MD simulation for structural validation and stability analysis. The predicted model was evaluated through RMSD (root mean square deviation), RMSF (root mean square fluctuation), SASA (solvent accessible surface area), and Rg (Radius of gyration).

### 4.2. Gene Network Analysis of ES

By examining gene networks, researchers may locate the most crucial genes and pathways associated with certain biological processes. This can facilitate by finding possible therapeutic targets or disease-related biomarkers. The protein network analysis of ES was carried out to check the possible involvement of DAX1 and observe other proteins which can also interact with ES. Therefore, the DisGeNET *Cytoscape* tool (https://www.disgenet.org/app (accessed on 28 March 2023)) was employed to construct the network of the proteins involved in ES.

### 4.3. Binding Site Prediction of DAX1

The binding pocket of proteins is most likely the active region where chemical compounds bind and perform their competitive and noncompetitive activity [17]. It consists of specific amino acid residues that form temporary bonds with the ligand (binding site) and residues that catalyze a reaction of that substrate (catalytic site). Prankweb (https://prankweb.cz/ (accessed on 4 April 2023)) an online server was utilized to explore the probability of amino acids involved in the formation of active binding sites. Furthermore, all the predicted binding pockets were evaluated based on scoring values, and the top pocket was selected based on the conformation and predicted score value. Therefore, the binding site was defined by the current selection method, and the docking sphere was contracted to get it restricted to our selected amino acid residues in the discovery studio.

### 4.4. Flavonoid Preparation

In cancer therapy, flavonoids have been investigated as potential chemo-preventive and chemotherapeutic agents, either as standalone treatments or in combination with conventional therapies. Furthermore, flavonoids have a variety of beneficial biochemical, antioxidant, anticancer, anti-allergic, anti-inflammatory, and antiviral properties related to various diseases [12,13]. The commercially available flavonoid compounds were retrieved from an online website Selleckchem (https://www.selleckchem.com/screening/Flavonoid-Compound-Library.html (accessed on 15 February 2023)) in SDF format. The flavonoid compounds were screened based on the RO5 and 132 were selected from the pool of 241 compounds. The selected flavonoids were further energy minimized and utilized for molecular docking studies.

### 4.5. Molecular Docking Analysis Using Discovery Studio

The leading approach for analyzing the interactions and conformations of ligands with target proteins is referred to as molecular docking [64]. By applying scoring functions, for instance, it is possible to forecast the association strength or binding patterns between two molecules by preferred orientation [11,65]. The CDocker tool of discovery studio was utilized to perform molecular docking of flavonoids against DAX1. The CDocker algorithm performs docking simulations and predicts the binding modes and strengths of small compounds to target protein by combining grid-based sampling, force field optimization, and scoring function assessment. It predicts two types of energies CDoker energy which represents the overall energy while the interaction energy includes the electrostatic and van der Waals interactions between the ligand and protein atoms in the docked complex, as well as other non-covalent interactions such as hydrogen bonding, pi-pi stacking, and hydrophobic interactions.

Therefore, for molecular docking studies, the grids box center values were adjusted as (X = −82.0604, Y = 0.5210, and Z = 50.5938) and the radius value of the grid was adjusted as 9.4825 for a better conformational position in the active region of the target protein. All the ligands (Flavonoids) were docked separately against DAX1 with default orientation and conformation 10 and 10 respectively. Therefore, top hits were selected as 05. The predicted docked complexes were evaluated on the basis of the lowest binding energy (Kcal/mol) values. The 3D graphical representations of the top five docked complexes were accomplished by UCSF Chimera 1.10.1 [66] and discovery studio (4.1), respectively.

### 4.6. Pharmacogenomics Analysis

The DisGeNET network (https://www.disgenet.org/ (accessed on 10 April 2023)) and the Drug Gene Interaction Database (DGIdb) (https://www.dgidb.org/ (accessed on 10 April 2023)) were used to generate an anticipated list of various disease-associated genes in order to construct the pharmacogenomics network model for the top ten chosen drugs [17]. In addition, all expected genes were thoroughly reviewed in the literature to assess their significance in ES. After sorting ES-related gene clusters and ES-associated gene clumps, the remaining genes were excluded from the data set.

### 4.7. Molecular Dynamic (MD) Simulation

The top 5 complexes luteolin, quercetin, kaempferol, flavopiridol, and epigallocatechin which manifest the lowest molecular docking energy (Kcal/mol) and good association with the genes involved in ES in pharmacogenomics analysis were brought to a 100ns MD simulation experiment.

The CHARMM36 force field was generated using the solution builder protocol of the CHARMM-GUI server (www.charm-gui.org (accessed on 12 April 2023)), and input files for MD simulations in GROMACS were prepared using the same methodology [67]. Therefore, 5 steps were carried out, in the first step the predicted 3D structure of DAX1 was uploaded in complex with the docked flavonoid compound (**1**), and in the second step TIP3P solution was employed to solvate the current model into a periodic, rectangular box that was extended 10 Å beyond each peptide’s atom. The ion plating method was selected as Monte-Carlo, the basic ion type was KCL and the ion concentration was adjusted to 0.15 by default. The counter ions were added up until the system was neutralized. In step three (solvation), The dimensions of the box along each axis (A, B, and C) were adjusted equal to 94 Å, which gives a total system size of approximately 830,584 cubic Angstroms. The crystal type was set as cubic in symmetry and the crystal angle values Alpha, Beta, and Gamma were set to 90 degrees, which are typical for cubic lattices. While, the box type was selected as rectangular. Furthermore, the Verlet cutoff technique with 10 Å was employed for electrostatic and Van der Waals interactions, while the LINCS algorithm was used to limit bonds. Additionally, the particle mesh Ewald (PME) method was used to calculate the electrostatic interactions. The solvated systems were subjected to the steepest descent energy minimization approach. After that (step four), Systems went through two equilibration phases. The NVT condition was applied to the systems before bringing them to equilibrium in the NPT condition and the temperature for the simulation was set at 30 °C. Therefore, at step five, for MD simulations in GROMACS, the CHARMM-GUI provides a format conversion Python script to create GROMACS topology (top) and parameter (itp) files. The GROMACS program (version 2019.3) was used with the Linux operating system to explore the structural behavior of protein and ligand complexes [68]. Production dynamics were conducted in GROMACS with a 2 fs time step, and for every picosecond, the coordinates were saved to a file for assessment.

## 5. Conclusions

In this computational study, the DAX1 protein was modeled to check the binding profile of flavonoids at its active site and proposed their therapeutic behavior in ES. Our results showed that flavonoids exhibited high compatibility against DAX1 by blocking the DAX1 active site with good conformation behavior. Furthermore, pharmacogenomics evaluation also confirms their good interactive profiles with genes (TNFRSF10B, CD34, and AR) having a direct relation with ES. In MD simulation results, the RMSD, hydrogen bond, and interaction energy graphs depicted good reliability of flavonoids against DAX1 protein. The MD results also explore that flavonoid compounds (luteolin, kaempferol, flavopiridol, and epigallocatechin) bind in the active region of the target protein which may indicate their inhibition behavior against DAX1. Taken together, luteolin, kaempferol, flavopiridol, and epigallocatechin exhibited good therapeutic potential against DAX1 and can be used as good competent drugs against ES after in vitro and in vivo evaluation.

## Figures and Tables

**Table 1:** Binding pocket residues prediction of DAX1 ranked based on the scoring values.

Name	Rank	Score	Residue
Pocket1	1	9.46	C_289 C_290 C_293 C_384 C_385 C_388 C_390 C_391 C_404 C_408 C_411 C_430 C_433 C_434 C_436 C_437
Pocket2	2	4.67	C_268 C_272 C_273 C_274 C_379 C_380 C_383 C_387 C_403 C_407 C_410
Pocket3	3	2.42	C_371 C_372 C_374 C_376 C_377 C_414 C_418 C_419 C_425
Pocket4	4	2.4	C_259 C_262 C_449 C_452 C_453 C_461 C_465
Pocket5	5	1.68	C_411 C_412 C_415 C_422 C_426 C_429
Pocket6	6	0.88	C_275 C_278 C_286 C_387 C_400 C_403

**Table 2:** The docking energy values (in kcal/mol) of flavonoids docked to Ewing sarcoma protein.

No.	Flavonoids	CDOCKER Energy (kcal/mol)	CDOCKER Interaction Energy (kcal/mol)
1	Luteolin	−48.5298	−45.7084
2	Quercetin	−45.4411	−53.2616
3	Kaempferol	−41.6088	−53.4284
4	Flavopiridol	−40.5963	−40.5658
5	Epigallocatechin	−40.3689	−38.8335
6	Taxifolin	−39.8385	−43.5986
7	Diosmetin	−39.809	−43.5293
8	Fisetin	−39.6941	−49.2916
9	7 8-Dihydroxyflavone	−39.5863	−40.1859
10	Scutellarein	−39.4315	−37.5446

**Table 3:** The predicted pharmacogenomics results.

Drug	Gene	Interaction Score	Disease	References
Luteolin	CYBB	1.29	Childhood osteosarcoma Carcinoma of lung Osteosarcoma of bone	[18,19]
	TNFRSF10B	0.55	Malignant neoplasm of the lung Osteosarcoma of bone Ewings sarcoma	[20,21,22]
	TYR	0.24	Neoplasm Metastasis Childhood Leukemia Sarcoma of Soft Tissue	[23,24,25]
	GPR35	0.16	Malignant Childhood Neoplasm Lung Carcinoma	[26,27]
	NFKB2	0.1	Childhood Lymphoma Bone Diseases	[28,29]
	RELA	0.08	Osteosarcoma of bone Malignant neoplasm of soft tissue	[30,31]
Quercetin	PKN1	0.78	Childhood Rhabdomyosarcoma	[32]
	GABPA	0.52	Bone tumorigenesis Carcinoma of lung Malignant neoplasm of soft tissue	[33,34,35,36]
	HSF1	0.52	Osteosarcoma of bone Childhood Osteosarcoma Childhood Leukemia	[37,38]
	CD34	0.39	Childhood Leukemia Ewing sarcoma Solitary fibrous tumor	[39,40,41]
	PKN3	0.39	Periodontal Diseases	[42]
	CDC25C	0.39	Carcinoma of lung	[43]
Kaempferol	CTDSP1	0.19	Carcinogenesis Breast Carcinoma	[44,45]
	NFKB2	0.18	Childhood Lymphoma Bone Diseases	[28,29]
	RELA	0.14	Osteosarcoma of bone Malignant neoplasm of soft tissue	[30,31]
	NFKB1	0.08	Malignant neoplasm of the lung Tumor Cell Invasion in the lungs Childhood Leukemia	[46,47,48]
	RACGAP1	0.05	Childhood Grade III Meningioma	[49]
Flavopiridol	AR	0.07	Ewing sarcoma Bone metastases	[50,51]
Epigallocatechin	ALB	5.89	Adenocarcinoma of lung Alzheimer’s Disease Osteosarcoma of bone	[52,53,54]
Taxifolin	POLH	0.05	Skin Carcinogenesis Skin Neoplasms	[55,56]
	POLB	0.05	Childhood Medulloblastoma	[57]
	CBX1	0.05	Myeloid Leukemia	[58]
Diosmetin	NFKB2	1.03	Childhood Lymphoma Bone Diseases	[28,29]
	RELA	0.81	Osteosarcoma of bone Malignant neoplasm of soft tissue	[30,31]
Fisetin	ALOX12	0.23	Childhood Lymphoma	[59]
	TOP2A	0.12	Soft tissue sarcoma	[60]
	TOP1	0.1	Childhood Leukemia	[61]

**Table 4:** The interaction energy of all five compounds in comparison with ifosfamide.

Sr. No.	Compounds Name	Interaction Energy		Total Energy
		Coulombic	Lenard Jones	
1	Luteolin	−80.7626	−101.066	−181.8286
2	Quercetin	−26.1085	−81.5833	−107.6918
3	Kaempferol	−75.8849	−91.6881	−167.573
4	Flavopiridol	−33.9002	−115.235	−149.1352
5	Epigallocatechin	−72.5864	−93.0951	−165.6815
6	Ifosfamide	−42.6356	−105.138	−147.7736

## Data Availability

All the data are contained within the manuscript and Supplementary Materials.

## Supplementary Materials

The following supporting information can be downloaded at: https://www.mdpi.com/article/10.3390/ijms24119332/s1.
