# Supplementary material for: Investigation of Flavonoid Scaffolds as DAX1 Inhibitors against Ewing Sarcoma through Pharmacoinformatic and Dynamic Simulation Studies
Source: Int J Mol Sci. 2023 May 26;24(11):9332. doi: 10.3390/ijms24119332 (PMC10253386; doi:10.3390/ijms24119332)
Supplement: Supplementary file 1 [file ijms-24-09332-s001.zip › ijms-2389426-supplementary.pdf]

Supporting Information

Investigation of flavonoid scaffolds as DAX1 inhibitors against Ewing sarcoma through pharmacoinformatic and dynamic simulation studies

Muhammad Yasir[1], Jinyoung Park[1], Eun-Taek Han[2], Won Sun Park[3], Jin-Hee Han[2], Yong-Soo Kwon[4], Hee-Jae Lee[1], Mubashir Hassan[5], Andrzej Kloczkowski[5] Wanjoo Chun[1,*]

[1]Department of Pharmacology, Kangwon National University School of Medicine, Chuncheon, 24341, Republic of Korea.
[2]Department of Medical Environmental Biology and Tropical Medicine, Kangwon National University School of Medicine, Chuncheon, 24341, Republic of Korea.
[3]Department of Physiology, Kangwon National University School of Medicine, Chuncheon, 24341, Republic of Korea.
[4]College of Pharmacy, Kangwon National University School of Medicine, Chuncheon, 24341, Republic of Korea.
[5]The Steve and Cindy Rasmussen Institute for Genomic Medicine at Nationwide Children's Hospital, Columbus, OH 43205, USA

Corresponding author: Dr. Wanjoo Chun, Department of Pharmacology Kangwon National University School of Medicine, Email: wchun@kangwon.ac.kr, Phone: 82-33-250-8853.

Table S1. ES network-associated genes are predicted in this table with association type and scoring values.

Sr no	Gene	Gene ID	Score	Association_Type	PMID	PMID Year
1	VIPR1	7433	0.01	Biomarker	10188714	1999
2	H4C13	8368	0.01	AlteredExpression	23185447	2012
3	SMUG1	23583	0.02	Biomarker	29100369	2017
4	KCMF1	56888	0.01	Biomarker	16314831	2006
5	CD99	4267	0.1	Biomarker	10492040	1999
6	ERG	2078	0.09	GeneticVariation	9049824	1997
7	FGF2	2247	0.02	Biomarker	15310753	2004
8	EWSR1	2130	0.1	GeneticVariation	17163154	2006
9	H4C15	554313	0.01	AlteredExpression	23185447	2012
10	H4C2	8366	0.01	AlteredExpression	23185447	2012
11	CD274	29126	0.01	AlteredExpression	29445891	2018
12	KCNN2	3781	0.01	Biomarker	23185447	2012
13	GRP	2922	0.01	Biomarker	31264078	2019
14	LIPI	149998	0.01	Biomarker	23291981	2013
15	H4C1	8359	0.01	AlteredExpression	23185447	2012
16	EWSR1	2130	0.1	GeneticVariation	27766786	2016
17	EWSR1	2130	0.1	Biomarker	25092916	2014
18	ERG	2078	0.09	AlteredExpression	22287547	2012
19	BMI1	648	0.01	Biomarker	18701473	2008
20	ERG	2078	0.09	AlteredExpression	23494411	2013
21	ECRG4	84417	0.01	Biomarker	23185447	2012
22	FLI1	2313	0.08	Biomarker	23494411	2013
23	TP63	8626	0.01	AlteredExpression	18769338	2008
24	EWSR1	2130	0.1	Biomarker	15659957	2005
25	CHGA	1113	0.01	Biomarker	10492040	1999
26	CD99	4267	0.1	GeneticVariation	16196395	2005
27	EWSR1	2130	0.1	Biomarker	21403840	2011
28	EWSR1	2130	0.1	GeneticVariation	22420726	2012
29	LDOC1	23641	0.01	Biomarker	23185447	2012
30	EWSR1	2130	0.1	GeneticVariation	21653923	2011
31	ENO2	2026	0.02	AlteredExpression	31264078	2019
32	EWSR1	2130	0.1	Biomarker	15877528	2005
33	FOLH1	2346	0.01	AlteredExpression	31524682	2020
34	EWSR1	2130	0.1	AlteredExpression	10565682	1999
35	EWSR1	2130	0.1	GeneticVariation	10888417	2000
36	PAX3	5077	0.01	AlteredExpression	9169092	1997
37	FLI1	2313	0.08	Biomarker	19411158	2009
38	EWSR1	2130	0.1	GeneticVariation	16314831	2006
39	EWSR1	2130	0.1	AlteredExpression	23760780	2013
40	EWSR1	2130	0.1	Biomarker	9178886	1997
41	KLRC4-KLRK1	1.01E+08	0.01	Biomarker	22355347	2012
42	FOXO4	4303	0.01	GeneticVariation	25007147	2014
43	EWSR1	2130	0.1	Biomarker	19144156	2009
44	CIC	23152	0.02	Biomarker	28864350	2017
45	EWSR1	2130	0.1	GeneticVariation	17610475	2007
46	HPR	3250	0.01	Biomarker	15289350	2004
47	CDKN2A	1029	0.02	AlteredExpression	11520274	2001
48	EWSR1	2130	0.1	Biomarker	31831298	2020
49	MYC	4609	0.01	Biomarker	19725831	2010
50	NR0B1	190	0.02	AlteredExpression	23185447	2012
51	ENO2	2026	0.02	GeneticVariation	10492040	1999
52	PIK3CB	5291	0.01	Biomarker	10582694	1999
53	H4C3	8364	0.01	AlteredExpression	23185447	2012

54	ABCC1	4363	0.01	AlteredExpression	16049968	2006
55	FLI1	2313	0.08	Biomarker	28296680	2017
56	EWSR1	2130	0.1	Biomarker	24293381	2014
57	RPE65	6121	0.01	AlteredExpression	18769338	2008
58	IL6	3569	0.01	Biomarker	25092916	2014
59	TP53	7157	0.01	GeneticVariation	25010205	2014
60	VIPR2	7434	0.01	AlteredExpression	10188714	1999
61	EWSR1	2130	0.1	AlteredExpression	22323082	2012
62	IGF1R	3480	0.02	Biomarker	10582694	1999
63	CD99	4267	0.1	Biomarker	19411158	2009
64	H4C9	8294	0.01	AlteredExpression	23185447	2012
65	IGF1R	3480	0.02	Biomarker	23760780	2013
66	SMARCB1	6598	0.01	Biomarker	19725831	2010
67	IGF1	3479	0.03	Biomarker	23760780	2013
68	EWSR1	2130	0.1	Biomarker	17762498	2007
69	CD99	4267	0.1	AlteredExpression	16096383	2005
70	STAG2	10735	0.01	GeneticVariation	25010205	2014
71	EWSR1	2130	0.1	Biomarker	12700668	2003
72	ERG	2078	0.09	Biomarker	24293381	2014
73	EWSR1	2130	0.1	GeneticVariation	16096383	2005
74	EWSR1	2130	0.1	GeneticVariation	21267687	2011
75	FLI1	2313	0.08	GeneticVariation	9704926	1998
76	BRCA2	675	0.01	GeneticVariation	25010205	2014
77	EWSR1	2130	0.1	Biomarker	19411158	2009
78	CTNNB1	1499	0.01	Biomarker	15266408	2004
79	ALK	238	0.01	Biomarker	11284039	2001
80	CD99	4267	0.1	AlteredExpression	16314831	2006
81	H4C11	8363	0.01	AlteredExpression	23185447	2012
82	SMUG1	23583	0.02	Biomarker	30508968	2018
83	ERG	2078	0.09	GeneticVariation	11471459	2001
84	EWSR1	2130	0.1	GeneticVariation	15049010	2004
85	EWSR1	2130	0.1	Biomarker	9836070	1998
86	PARP1	142	0.01	Biomarker	22287547	2012
87	ASAH1	427	0.01	AlteredExpression	15289350	2004
88	CD99	4267	0.1	AlteredExpression	23760780	2013
89	CD99	4267	0.1	Biomarker	10416880	1999
90	CD99	4267	0.1	AlteredExpression	21267687	2011
91	ERG	2078	0.09	Biomarker	10561219	1999
92	FLI1	2313	0.08	Biomarker	23185447	2012
93	EWSR1	2130	0.1	AlteredExpression	24312454	2013
94	GFAP	2670	0.01	AlteredExpression	28296680	2017
95	H4C5	8367	0.01	AlteredExpression	23185447	2012
96	S100A1	6271	0.01	GeneticVariation	10492040	1999
97	ERG	2078	0.09	Biomarker	23185447	2012
98	FLII	2314	0.03	Biomarker	28296680	2017
99	EWSR1	2130	0.1	Biomarker	25755803	2015
100	EWSR1	2130	0.1	Biomarker	17272319	2007
101	EWSR1	2130	0.1	Biomarker	27627705	2016
102	CEACAM5	1048	0.01	Biomarker	11508817	2001
103	EWSR1	2130	0.1	GeneticVariation	16196395	2005
104	CDK4	1019	0.01	Biomarker	9393981	1997
105	EWSR1	2130	0.1	Biomarker	8084618	1994
106	CD99	4267	0.1	Biomarker	20182342	2010
107	NPL	80896	0.01	GeneticVariation	25733708	2015
108	NUTM1	256646	0.01	Biomarker	30453921	2018
109	EWSR1	2130	0.1	GeneticVariation	9704926	1998
110	CAV1	857	0.02	AlteredExpression	23185447	2012
111	EWSR1	2130	0.1	GeneticVariation	10078922	1999

112	PIK3CD	5293	0.01	Biomarker	10582694	1999
113	EWSR1	2130	0.1	GeneticVariation	16052075	2005
114	EWSR1	2130	0.1	Biomarker	15930281	2005
115	CIC	23152	0.02	GeneticVariation	25007147	2014
116	BCOR	54880	0.01	GeneticVariation	28864350	2017
117	EWSR1	2130	0.1	Biomarker	25010205	2014
118	FLI1	2313	0.08	GeneticVariation	9049825	1997
119	EWSR1	2130	0.1	GeneticVariation	9049825	1997
120	B3GAT1	27087	0.01	Biomarker	10492040	1999
121	ERG	2078	0.09	Biomarker	9836070	1998
122	EWSR1	2130	0.1	Biomarker	11471459	2001
123	EWSR1	2130	0.1	Biomarker	16721801	2006
124	EWSR1	2130	0.1	AlteredExpression	10976720	2000
125	EWSR1	2130	0.1	Biomarker	10949935	2000
126	EWSR1	2130	0.1	AlteredExpression	18591936	2008
127	EWSR1	2130	0.1	Biomarker	15491164	2004
128	CAV1	857	0.02	Biomarker	21106507	2010
129	EWSR1	2130	0.1	Biomarker	28296680	2017
130	EWSR1	2130	0.1	Biomarker	11284039	2001
131	CD99	4267	0.1	Biomarker	28864350	2017
132	FLII	2314	0.03	GeneticVariation	9049824	1997
133	MYCN	4613	0.01	Biomarker	19725831	2010
134	EWSR1	2130	0.1	Biomarker	19725831	2010
135	UVRAG	7405	0.01	AlteredExpression	18769338	2008
136	EWSR1	2130	0.1	Biomarker	12908547	2003
137	CASP3	836	0.01	AlteredExpression	10582694	1999
138	EWSR1	2130	0.1	Biomarker	22287547	2012
139	WRN	7486	0.01	GeneticVariation	31831298	2020
140	EWSR1	2130	0.1	Biomarker	10088552	1999
141	EWSR1	2130	0.1	Biomarker	16740692	2006
142	EWSR1	2130	0.1	GeneticVariation	20513536	2010
143	FLII	2314	0.03	GeneticVariation	9049825	1997
144	EWSR1	2130	0.1	AlteredExpression	23185447	2012
145	DLEU7	220107	0.01	Biomarker	10492040	1999
146	CDKN2A	1029	0.02	Biomarker	18701473	2008
147	ABCB1	5243	0.01	AlteredExpression	16049968	2006
148	MTAP	4507	0.01	Biomarker	29243509	2018
149	IGFBP3	3486	0.01	Biomarker	23185447	2012
150	VIP	7432	0.01	Biomarker	10188714	1999
151	H4C12	8362	0.01	AlteredExpression	23185447	2012
152	EWSR1	2130	0.1	Biomarker	16157025	2005
153	EWSR1	2130	0.1	Biomarker	18088234	2008
154	EWSR1	2130	0.1	GeneticVariation	17557870	2007
155	H4C6	8361	0.01	AlteredExpression	23185447	2012
156	COMMD3-BMI1	1.01E+08	0.01	Biomarker	18701473	2008
157	SPARC	6678	0.01	AlteredExpression	21106507	2010
158	EWSR1	2130	0.1	GeneticVariation	10493837	1999
159	FLI1	2313	0.08	GeneticVariation	19144156	2009
160	EWSR1	2130	0.1	AlteredExpression	23494411	2013
161	EWSR1	2130	0.1	AlteredExpression	25688366	2015
162	EWSR1	2130	0.1	GeneticVariation	27180056	2016
163	CD99	4267	0.1	Biomarker	28296680	2017
164	PIK3CA	5290	0.01	Biomarker	10582694	1999
165	H4C4	8360	0.01	AlteredExpression	23185447	2012
166	PDGFC	56034	0.01	Biomarker	12700668	2003
167	TGFBR2	7048	0.01	Biomarker	23185447	2012
168	IGF1	3479	0.03	Biomarker	8054516	1994

169	EWSR1	2130	0.1	Biomarker	20308673	2010
170	EWSR1	2130	0.1	GeneticVariation	29581854	2018
171	CCND1	595	0.01	Biomarker	9393981	1997
172	KRT20	54474	0.01	Biomarker	20182342	2010
173	EWSR1	2130	0.1	Biomarker	11037342	2000
174	PIK3CG	5294	0.01	Biomarker	10582694	1999
175	SST	6750	0.01	AlteredExpression	10231868	1999
176	EWSR1	2130	0.1	Biomarker	16556028	2005
177	CKAP4	10970	0.01	AlteredExpression	18769338	2008
178	EWSR1	2130	0.1	Biomarker	10492040	1999
179	EWSR1	2130	0.1	AlteredExpression	17438102	2007
180	EWSR1	2130	0.1	GeneticVariation	20473914	2011
181	IGF1	3479	0.03	Biomarker	10582694	1999
182	FEV	54738	0.01	GeneticVariation	31831298	2020
183	FLI1	2313	0.08	Biomarker	9836070	1998
184	EWSR1	2130	0.1	Biomarker	22429598	2012
185	H4C14	8370	0.01	AlteredExpression	23185447	2012
186	BCL2	596	0.01	Biomarker	30348635	2019
187	EWSR1	2130	0.1	Biomarker	22879388	2012
188	H3P10	1.15E+08	0.01	AlteredExpression	18701473	2008
189	FGF2	2247	0.02	PosttranslationalModification	18263590	2008
190	H4C8	8365	0.01	AlteredExpression	23185447	2012
191	CD34	947	0.01	Biomarker	17369866	2007
192	S100B	6285	0.01	GeneticVariation	10492040	1999
193	DHX9	1660	0.01	Biomarker	16740692	2006
194	ERG	2078	0.09	Biomarker	21519790	2011
195	NR0B1	190	0.02	Biomarker	18591936	2008
196	EWSR1	2130	0.1	GeneticVariation	9049824	1997
197	EWSR1	2130	0.1	GeneticVariation	21106507	2010
198	H4-16	121504	0.01	AlteredExpression	23185447	2012
199	SLFN11	91607	0.01	AlteredExpression	25733708	2015
200	C17orf97	400566	0.01	Biomarker	20182342	2010
201	EWSR1	2130	0.1	Biomarker	11519035	2001
202	KLRK1	22914	0.01	Biomarker	22355347	2012

Table S2. Molecular docking score of initial 132 compounds with PubChem CID and respective 2D images.

Sr no	Compounds	CDocker energy	CDocker interaction energy	PubChem CID	2D images
1	Luteolin	-48.5298	-45.7084	5280445	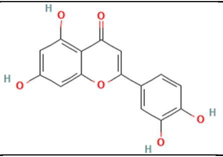
2	Quercetin	-45.4411	-53.2616	5280343	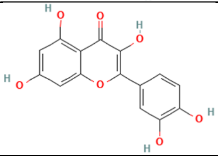
3	Kaempferol	-41.6088	-53.4284	5280863	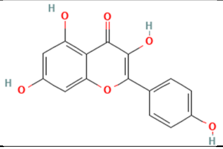
4	Flavopiridol	-40.5963	-40.5658	5287969	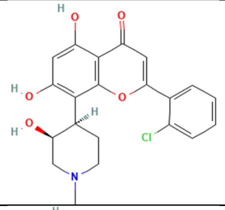
5	Epigallocatechin	-40.3689	-38.8335	72277	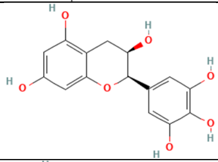
6	Taxifolin	-39.8385	-43.5986	439533	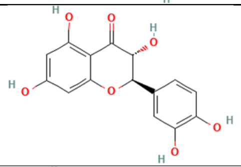
7	Diosmetin	-39.809	-43.5293	5281612	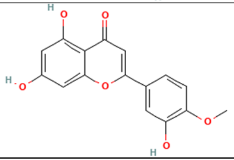
8	Fisetin	-39.6941	-49.2916	5281614	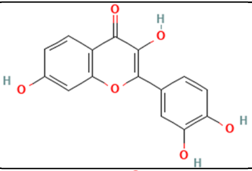
9	7_8-Dihydroxyflavone	-39.5863	-40.1859	1880	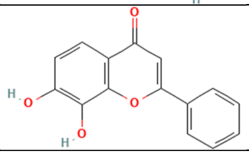

10	Scutellarein	-39.4315	-37.5446	5281697	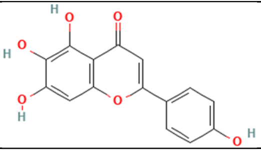
11	Phloretin	-39.1557	-43.8202	4788	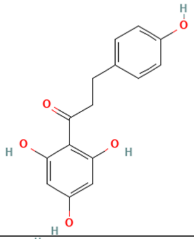
12	Apigenin	-38.7233	-41.5549	5280443	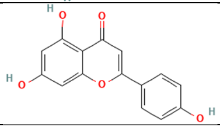
13	S9287_Genkwanin	-38.0676	-43.5516	5281617	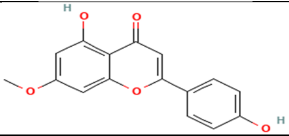
14	Baicalein	-37.931	-35.7685	5281605	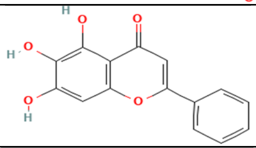
15	Genistein	-37.9187	-42.312	5280961	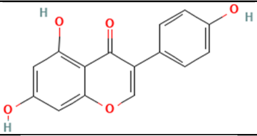
16	Hesperetin	-37.2704	-43.6884	72281	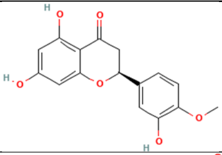
17	Daidzein	-37.1637	-38.8963	5281708	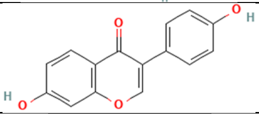
18	S3836_6-Gingerol	-36.8494	-39.1428	442793	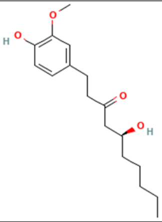
19	Naringenin	-36.7988	-41.7884	932	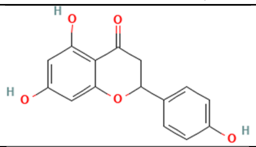
20	Equol	-36.6572	-41.799	91469	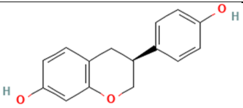

21	S5380_7-Hydroxyflavone	-36.2863	-38.7844	5281894	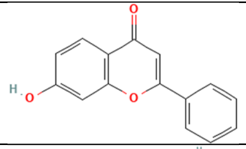
22	Loureirin_B	-36.0644	-45.1739	189670	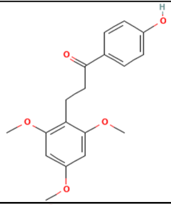
23	2-D08	-36.0278	-34.6996	22507438	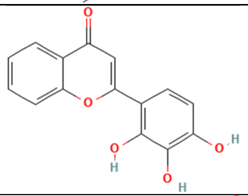
24	(__)-Equol	-34.5731	-39.712	382975	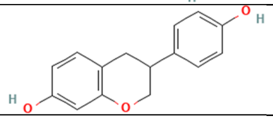
25	Loureirin_A	-34.3043	-41.8647	5319081	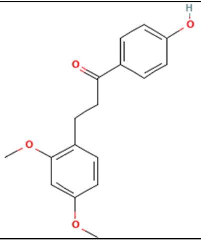
26	S9122_Tectorigenin	-33.8531	-43.1069	5281811	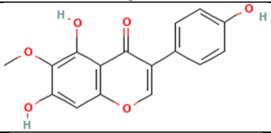
27	S9123_Eriodictyol	-33.1403	-38.8122	440735	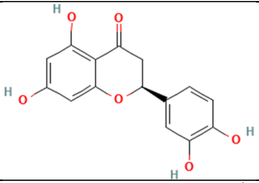
28	S9038_Calycosin	-33.1064	-41.0947	5280448	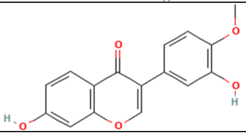
29	Hispidulin	-32.7102	-39.7315	5281628	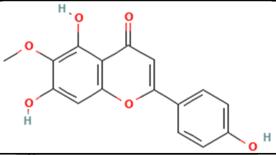
30	S9378_4_5-Dihydroxyflavone	-32.679	-34.9683	165521	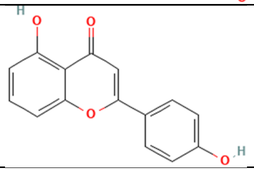

31	S4937_4_-Hydroxychalcone	-32.4074	-38.3929	5282361	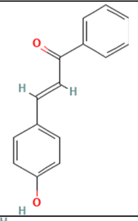
32	S9048_(+)-Gallocatechin	-32.385	-37.7539	65084	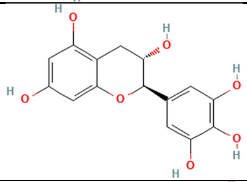
33	S9107_Glycitein	-32.0008	-41.5553	5317750	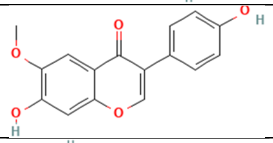
34	S9110_Morin	-30.6406	-35.5764	5281670	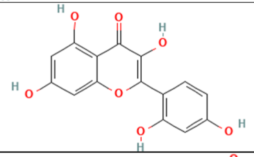
35	Biochanin_A	-30.6199	-38.0726	5280373	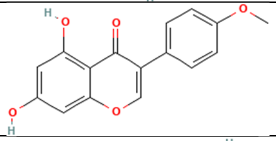
36	S9440_Naringenin_chalcone	-30.3047	-40.3001	5280960	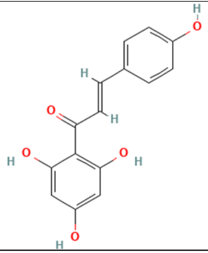
37	Pectolinarigenin	-30.1523	-42.3097	5320438	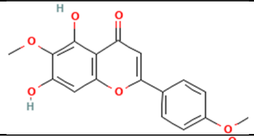
38	Formononetin	-29.7956	-36.3391	5280378	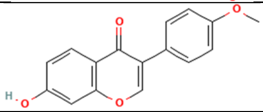
39	S9437_Echinatin	-29.7129	-39.4753	6442675	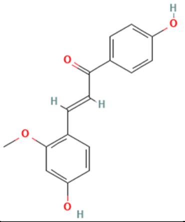

40	S9111_Isorhamnetin	-29.678	-46.7164	5281654	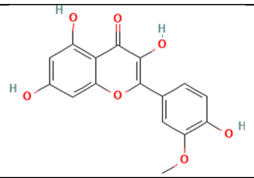
41	S5548_7-Hydroxy-4-chromone	-29.2685	-27.2261	5409279	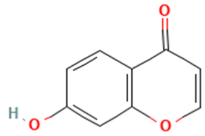
42	S9205_Hydroxygenkwanin	-28.9535	-34.8541	5318214	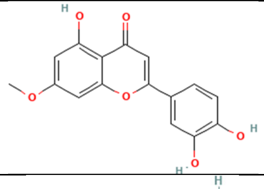
43	Butein	-28.6049	-36.3643	5281222	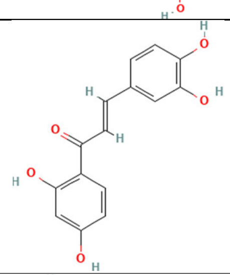
44	S4723_(-)Epicatechin	-28.2159	-32.8692	72276	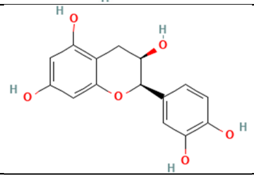
45	(+)-Catechin	-27.894	-35.0282	9064	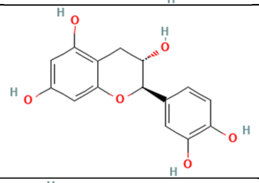
46	Farrerol	-27.7669	-33.6903	91144	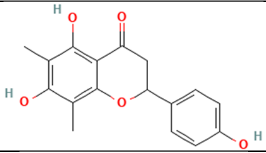
47	Galangin_3-methyl_ether	-27.633	-37.3358	—	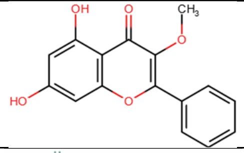
48	S3846_Eupatilin	-26.7569	-43.7569	5273755	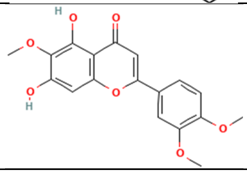

49	S9043_6-Shogaol	-26.7403	-34.7972	5281794	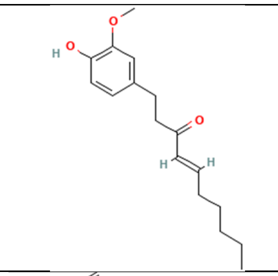
50	Licochalcone_A	-26.2474	-47.0011	5318998	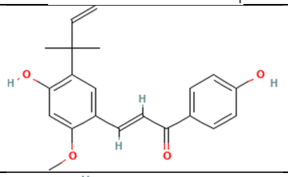
51	Chrysin	-25.2715	-30.9232	5281607	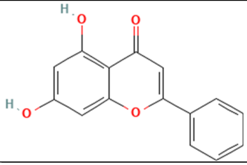
52	Pinocembrin	-25.2236	-33.2761	68071	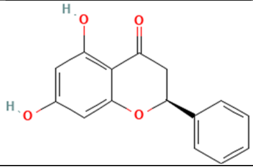
53	Acacetin	-25.073	-33.1322	5280442	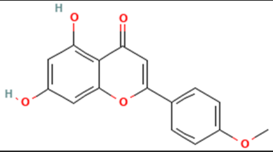
54	Corylin	-24.8121	-43.3809	5316097	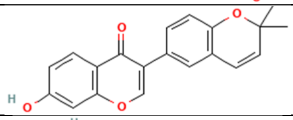
55	Hispidulin	-24.5561	-33.6978	5281628	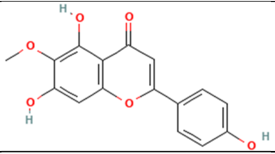
56	3-Hydroxyphenylacetic	-24.3823	-25.661	12122	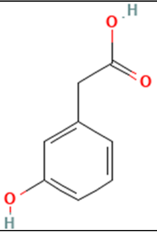
57	Isosakuranetin	-24.0802	-32.0848	160481	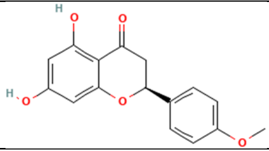

58	4_7-Dimethoxy-5-Hydroxyflavone	-24.0673	-33.853	11511802	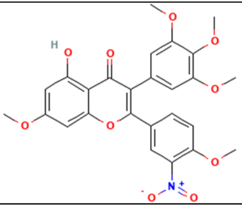
59	Phenoxodiol	-23.9828	-37.3348	219100	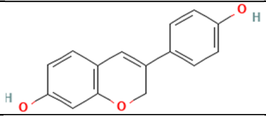
60	S3929_Liquiritigenin	-23.8663	-29.0568	114829	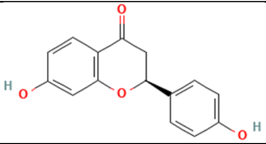
61	S3877_Lysionotin	-23.1191	-43.5881	160921	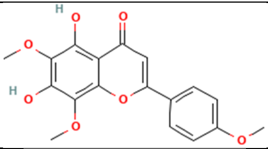
62	S4743_Wogonin	-23.0372	-31.7747	5281703	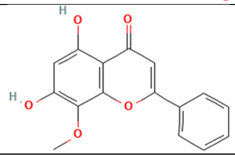
63	S3879_kaempferide	-23.0022	-38.0133	5281666	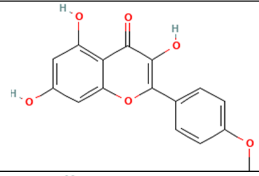
64	S9204_Oroxylin_A	-22.9916	-32.1963	5320315	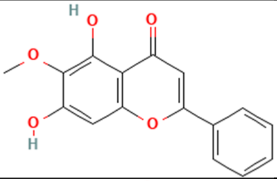
65	S3634_6-Hydroxyflavone	-22.9186	-26.7713	72279	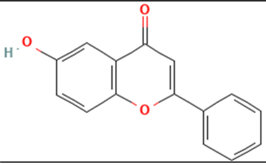
66	Isoliquiritigenin	-22.8938	-31.9716	638278	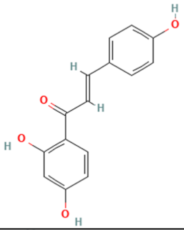
67	Tectochrysin	-22.4827	-29.7013	5281954	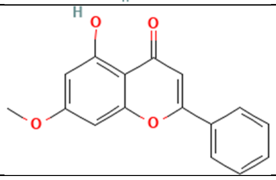

68	Ipriflavone	-22.1278	-32.7714	3747	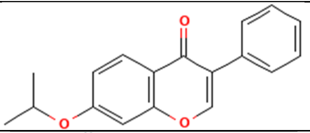
69	S5529_Galangin	-21.6262	-34.3366	5281616	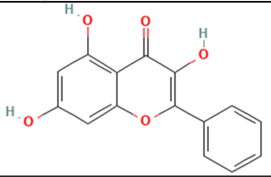
70	S3875_Alpinetin	-20-.9374	-32.1682	154279	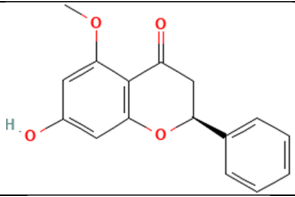
71	S4790_3-Hydroxyflavone	-20.6897	-33.1733	11349	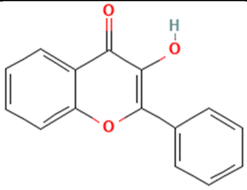
72	S3679_Flavanone	-20.6088	-26.3532	10251	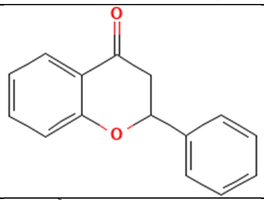
73	4_5_7-Trimethoxyflavone	-20.4558	-35.4312	79730	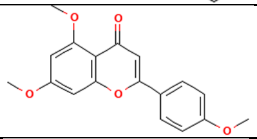
74	S3967_Flavone	-20.4544	-25.3732	10680	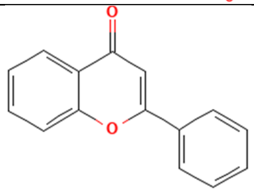
75	Anhydroicaritin	-20.3019	-35.8563	14583584	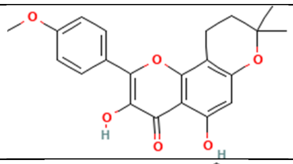
76	S3942_Cardamonin	-19.9796	-33.2601	641785	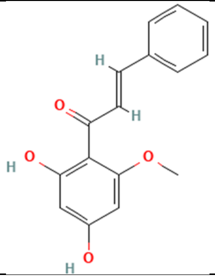

77	4_7-Dimethoxyisoflavone	-19.7538	-32.5723	136419	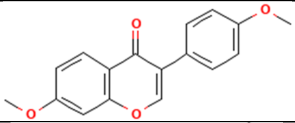
78	7-Methoxyisoflavone	-19.4308	-29.4646	638006	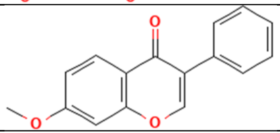
79	5_7-Dimethoxyflavone	-19.3502	-31.9309	88881	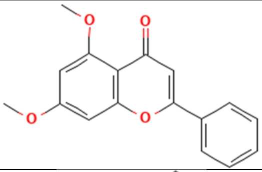
80	S4763_4-Hydroxychalcone	-19.2626	-27.6329	5282361	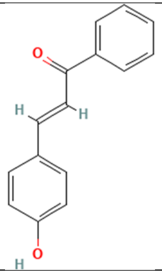
81	S4782_5-methoxyflavone	-19.233	-29.2379	94525	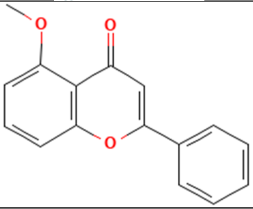
82	Xanthone	-18.4706	-23.3239	7020	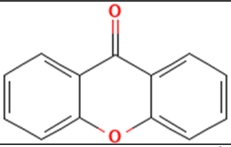
83	5-Methyl-7-methoxyisoflavone	-18.2903	-30.6319	2734290	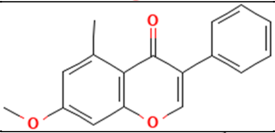
84	S9121_Irigenin	-17.9964	-38.2519	5464170	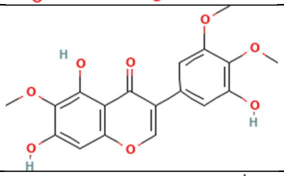
85	S5600_Flavokawain_A	-17.9156	-36.8903	5355469	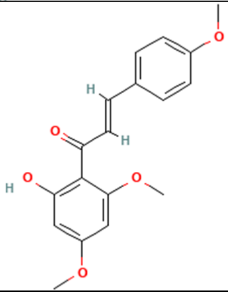

86	2_-Hydroxy-2-methoxychalcone	-17.7422	-29.9289	466234	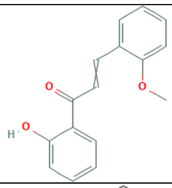
87	4_-Methoxychalcone	-17.1169	-28.8342	641818	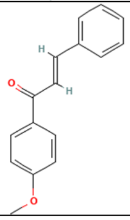
88	S9286_Neobavaisoflavone	-16.9939	-46.5358	5320053	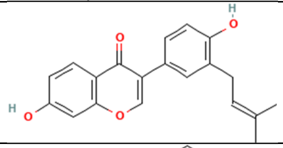
89	Chalcone	-16.9671	-25.5922	637760	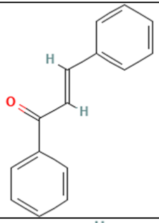
90	S4765_Syringaldehyde	-16.0628	-23.4786	8655	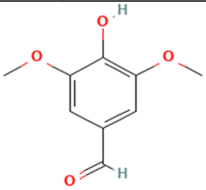
91	2_-Hydroxyacetophenone	-16.0206	-18.7215	68490	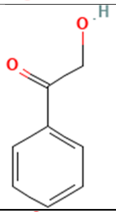
92	Sappanone_A	-15.9914	-35.2132	9817274	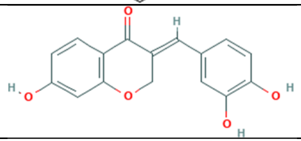
93	S3763_Cinnamaldehyde	-15.7019	-19.3291	637511	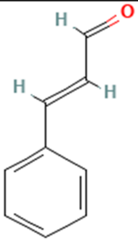

94	S9412_Flavokawain_B	-15.6029	-30.2809	5356121	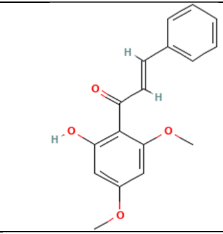
95	S9080_Anhydroicaritin	-15.4074	-51.3542	14583584	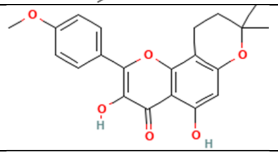
96	S3786_Glabridin	-15.1108	-34.942	124052	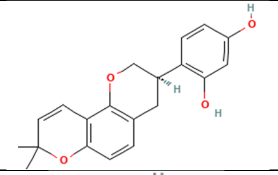
97	S4940_Maltol	-14.5467	-30.2049	8369	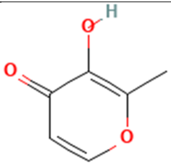
98	Flavoxate_HCL	-14.4489	-38.6933	441345	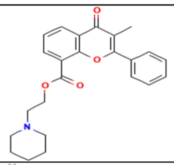
99	S9288_Casticin	-13.2626	-35.6998	5315263	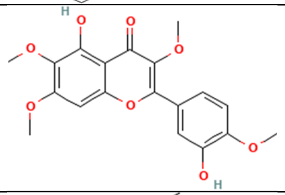
100	1_3_5-Trimethoxybenzene	-13.0566	-23.3705	69301	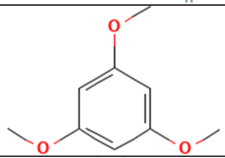
101	Deguelin	-11.6169	-39.3658	107935	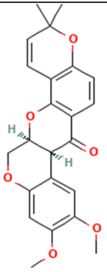

102	Hematoxylin	-11.5116	-32.9339	442514	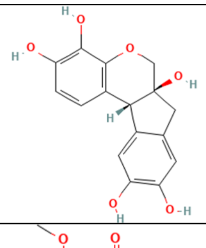
103	5_6_7-Trimethoxyflavone	-11.4458	-30.9628	442583	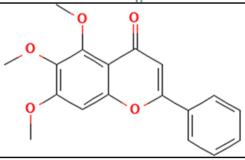
104	S5158_alpha-Naphthoflavone	-11.4145	-26.1406	11790	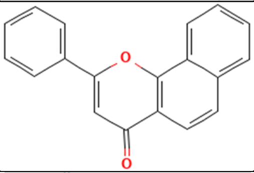
105	S9421_Demethylnobiletin	-10.6472	-37.7838	358832	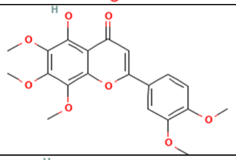
106	Artemitin	-10.0914	-41.2204	5320351	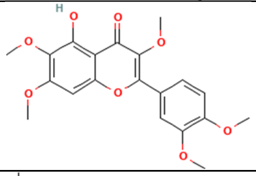
107	S5469_Bavachin	-9.75184	-36.2419	14236566	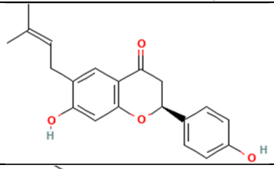
108	S9227_Sinensetin	-9.53312	-35.9903	145659	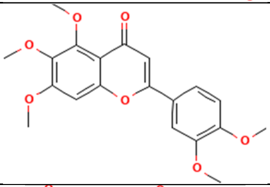
109	S9428_Brazilin	-8.84052	-33.2891	73384	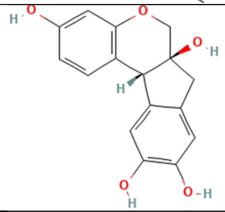
110	S9113_Ononin	-8.74301	-43.518	442813	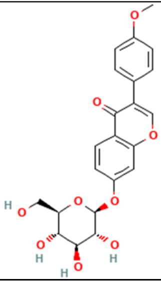

111	S3878_Bavachinin	-7.31819	-37.2092	10337211	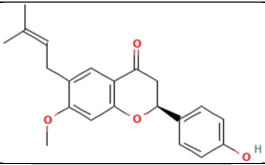
112	S5456_Isobavachalcone	-7.04957	-37.3183	5281255	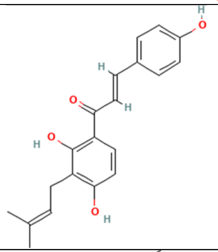
113	Khellin	-6.89661	-28.7396	3828	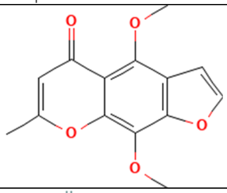
114	p276-00	-6.61931	-34.8407	23643975	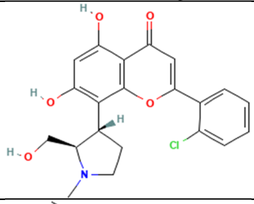
115	Nobiletin	-5.99673	-39.2851	72344	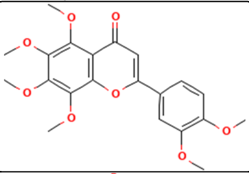
116	S9256_Isobavachin	-5.60828	-30.7641	193679	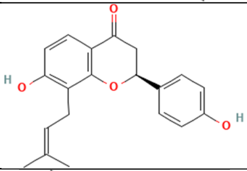
117	S9270_Dracohodin_perochlorate	-5.44955	-29.8582	74787691	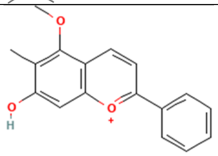
118	Tangeretin	-4.47936	-34.1213	68077	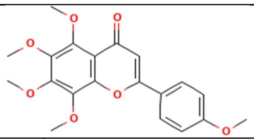
119	S9070_Isoxanthohumol	-4.20233	-35.8538	513197	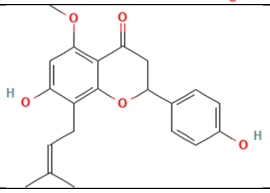

120	S9409_Kavain	-3.93303	-27.7681	5281565	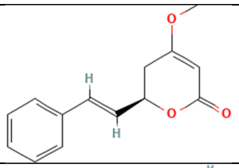
121	Xanthohumol	-3.82703	-37.5482	639665	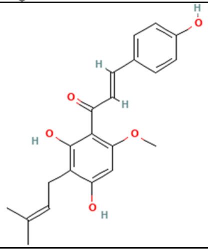
122	S9028_Cimifugin	-2.36416	-36.3477	441960	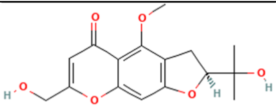
123	S9053_Irisflorentin	5.22834	-39.086	170569	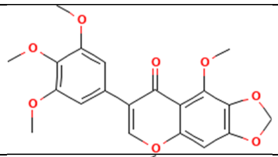
124	Rotenone	8.60649	-37.1983	6758	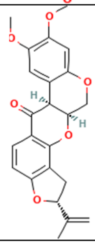
125	S9387_Maackiain	12.5138	-30.6821	91510	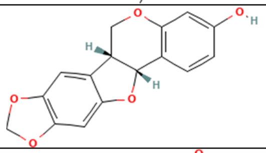
126	5a-Pregnane-3_20-dione	15.8379	-34.506	92810	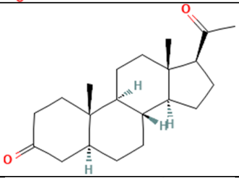
127	Medroxyprogesterone_acetate	17.8314	-36.4916	6279	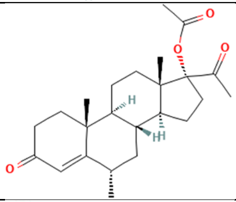
128	S3784_Obacunone	21.4605	-39.9202	119041	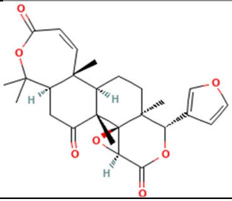

129	17-Hydroxyprogesterone	24.8666	-34.6295	6238	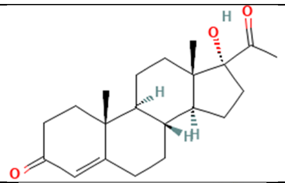
130	S3635_Medroxyprogesterone	24.9057	-33.9816	10631	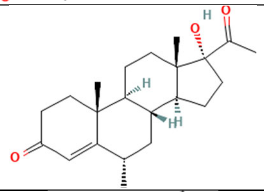
131	S9425_Trifolirhizin	26.1629	-43.7232	442827	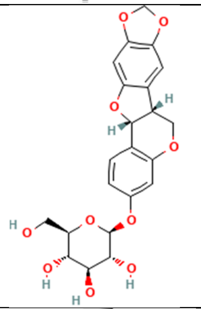
132	S3800_Lycorine_hydrochloride	39.801	-28.2448	164943	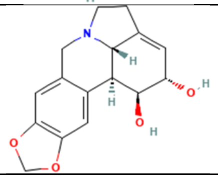

Table S3. Chemoinformatic properties of screened compounds

Name	H_acceptor	H_Donor	M_weight	Log_P	Rotatable_bonds	Aromatic_rings
Genistein	5	3	270.237	2.14	1	2
Daidzein	4	2	254.238	2.382	1	2
Silibinin	10	5	482.436	2.592	4	3
Silymarin	10	5	482.436	2.592	4	3
Tangeretin	7	0	372.369	3.054	6	2
Xanthone	2	0	196.201	2.964	0	2
Biochanin A	5	2	284.263	2.366	2	2
Diosmetin	6	3	300.263	2.394	2	2
Hematoxylin	6	5	302.279	1.691	0	2
Quercetin	7	5	302.236	1.63	1	2
Naringenin	5	3	272.253	2.373	1	2
Apigenin	5	3	270.237	2.41	1	2
Ipriflavone	3	0	280.318	3.576	3	2
Baicalein	5	3	270.237	2.41	1	2
Chrysin	4	2	254.238	2.652	1	2
Fisetin	6	4	286.236	1.872	1	2
Formononetin	4	1	268.264	2.608	2	2
Hesperetin	6	3	302.279	2.357	2	2
Kaempferol	6	4	286.236	1.872	1	2
Luteolin	6	4	286.236	2.168	1	2
Nobiletin	8	0	402.395	3.038	7	2
Phloretin	5	4	274.269	2.757	4	2
Isoliquiritigenin	4	3	256.253	2.975	3	2
S3942 Cardamonin	4	2	270.28	3.201	4	2
Medroxyprogesterone acetate	4	0	386.524	3.755	3	0
Flavopiridol hydrochloride	6	3	438.301	3.49	2	2
Equol	3	2	242.27	3.211	1	2
Daidzin	9	5	416.378	0.452	4	2
Taxifolin (Dihydroquercetin)	7	5	304.252	1.479	1	2
Flavopiridol hydrochloride	6	3	401.84	3.108	2	2
17-Hydroxyprogesterone	3	1	330.461	3.124	1	0
S4723 Epicatechin	6	5	290.268	2.021	1	2
S4743 Wogonin	5	2	284.263	2.636	2	2
S3634 6-Hydroxyflavone	3	1	238.238	2.894	1	2
S3635 Medroxyprogesterone	3	1	344.488	3.376	1	0
S3679 Flavanone	2	0	224.255	3.099	1	2
Rotenone	6	0	394.417	3.93	3	2
S3941 Pinocembrin	4	2	256.253	2.615	1	2
S3942 Cardamonin	4	2	270.28	3.201	4	2
S3825 Glycitin	10	5	446.404	0.436	5	2
S3836 6-Gingerol	4	2	294.386	3.638	10	1
S3784 Obacunone	7	0	454.512	2.715	1	1
S3786 Glabridin	4	2	324.37	3.999	1	2
4',7-Dimethoxy-5-Hydroxyflavone	5	1	298.29	2.861	3	2
S3929 Liquiritigenin	4	2	256.253	2.615	1	2
S3930 Liquiritin	9	5	418.394	0.686	4	2
S3846 Eupatilin	7	2	344.315	2.603	4	2
S3875 Alpinetin	4	1	270.28	2.841	2	2
S3877 Lysionotin	7	2	344.315	2.603	4	2
S3878 Bavachinin	4	1	338.397	4.698	4	2
S3800 Lycorine hydrochloride	5	2	323.771	1.095	0	1
p276-00	6	3	438.301	3.49	3	2

S3910 4',7-Dimethoxyisoflavone	4	0	282.291	2.833	3	2
S3903 Lycorine	5	2	287.31	0.713	0	1
S4790 3-Hydroxyflavone	3	1	238.238	2.598	1	2
S4782 5-methoxyflavone	3	0	252.265	3.12	2	2
S4763 4-Hydroxychalcone	2	1	224.255	3.46	3	2
S4765 Syringaldehyde	4	1	182.173	1.314	3	1
S3967 Flavone	2	0	222.239	3.136	1	2
S3879 kaempferide	6	3	300.263	2.098	2	2
S4940 Maltol	3	1	126.11	-0.222	0	0
S4937 4'-Hydroxychalcone	2	1	224.255	3.46	3	2
Flavoxate HCL	5	0	427.921	4.876	6	2
Butein	5	4	272.253	2.734	3	2
Xanthohumol	5	3	354.396	4.816	6	2
S3763 Cinnamaldehyde	1	0	132.159	1.949	2	1
Licochalcone A	4	2	338.397	4.667	6	2
7,8-Dihydroxyflavone	4	2	254.238	2.652	1	2
Deguelin	6	0	394.417	3.567	2	2
S4722 (+)-Catechin	6	5	290.268	2.021	1	2
2-D08	5	3	270.237	2.41	1	2
S5318 Acacetin	5	2	284.263	2.636	2	2
S5158 alpha-Naphthoflavone	2	0	272.297	4.044	1	3
S5380 7-Hydroxyflavone	3	1	238.238	2.894	1	2
S5456 Isobavachalcone	4	3	324.37	4.832	5	2
S5469 Bavachin	4	2	324.37	4.472	3	2
S5529 Galangin	5	3	270.237	2.114	1	2
S9378 4',5-Dihydroxyflavone	4	2	254.238	2.652	1	2
S9387 Maackiain	5	1	284.263	2.467	0	2
S9409 Kavain	3	0	230.259	2.218	3	1
S9412 Flavokawain B	4	1	284.307	3.427	5	2
S9421 Demethylnobiletin	8	1	388.368	2.812	6	2
S9425 Trifolirhizin	10	4	446.404	0.537	3	2
S9428 Brazilin	5	4	286.279	1.933	0	2
S9437 Echinatin	4	2	270.28	3.201	4	2
S9438 Isosakuranetin	5	2	286.279	2.599	2	2
S9440 Naringenin chalcone	5	4	272.253	2.734	3	2
Chalcone	1	0	208.255	3.701	3	2
4'-Methoxychalcone	2	0	238.281	3.685	4	2
2'-Hydroxy-2-methoxychalcone	3	1	254.281	3.443	4	2
S9028 Cimifugin	6	2	306.311	1.127	3	1
S9043 6-Shogaol	3	1	276.371	4.717	9	1
S9053 Irisflorentin	8	0	386.352	2.569	5	2
S9070 Isoxanthohumol	5	2	354.396	4.456	4	2
S9080 Anhydroicaritin	6	3	368.38	3.954	4	2
S9088 Calycosin-7-O-beta-D-glucoside	10	5	446.404	0.436	5	2
S9107 Glycitein	5	2	284.263	2.366	2	2
S9110 Morin	7	5	302.236	1.63	1	2
S9111 Isorhamnetin	7	4	316.262	1.856	2	2
S9113 Ononin	9	4	430.405	0.678	5	2
S9120 Scutellarein	6	4	286.236	2.168	1	2
S9121 Irigenin	8	3	360.315	2.091	4	2
S9122 Tectorigenin	6	3	300.263	2.124	2	2
S9123 Eriodictyol	6	4	288.252	2.131	1	2
S9139 5-Methyl-7-methoxyisoflavone	3	0	266.291	3.336	2	2

S9173 Sec-O-Glucosylhamaudol	10	5	438.425	0.042	3	1
S9204 Oroxylin A	5	2	284.263	2.636	2	2
S9205 Hydroxygenkwanin	6	3	300.263	2.394	2	2
S9227 Sinensetin	7	0	372.369	3.054	6	2
S9250 Isosilybin	10	5	482.436	2.592	4	3
S9256 Isobavachin	4	2	324.37	4.472	3	2
S9270 Dracohodin perochlorate	7	1	366.75	1.785	2	3
S9286 Neobavaisoflavone	4	2	322.355	4.239	3	2
S9287 Genkwanin	5	2	284.263	2.636	2	2
S9288 Casticin	8	2	374.341	2.291	5	2
S9298 Corylin	4	1	320.339	3.412	1	2
S5548 7-Hydroxy-4-chromone	3	1	162.142	1.146	0	1
S5600 Flavokawain A	5	1	314.333	3.41	6	2
S9038 Calycosin	5	2	284.263	2.366	2	2
S9328 5,6,7-Trimethoxyflavone	5	0	312.317	3.087	4	2
S9375 2'-Hydroxyacetophenone	2	1	136.148	1.327	1	1
Farrerol	5	3	300.306	3.346	1	2
1,3,5-Trimethoxybenzene	3	0	168.19	1.78	3	1
Khellin	5	0	260.242	2.172	2	2
Equol	3	2	242.27	3.211	1	2
5a-Pregnane-3,20-dione	2	0	316.478	3.689	1	0
5,7-Dimethoxyflavone	4	0	282.291	3.103	3	2
Loureirin A	4	1	286.322	3.45	6	2
Loureirin B	5	1	316.348	3.434	7	2
Pectolinarigenin	6	2	314.289	2.619	3	2
Tectochrysin	4	1	268.264	2.878	2	2
Hispidulin	6	3	300.263	2.394	2	2
Galangin 3-methyl ether	5	2	284.263	2.34	2	2
Phenoxodiol (Haginin E)	3	2	240.254	3.029	1	2
4',5,7-Trimethoxyflavone	5	0	312.317	3.087	4	2
7-Methoxyisoflavone	3	0	252.265	2.85	2	2
3-Hydroxyphenylacetic acid	3	2	152.147	1.252	2	1
Anhydroicaritin	6	2	368.38	3.437	2	2
Sappanone A	5	3	284.263	2.673	1	2
Trans-Chalcone	1	0	208.255	3.701	3	2
Artemitin	8	1	388.368	2.516	6	2

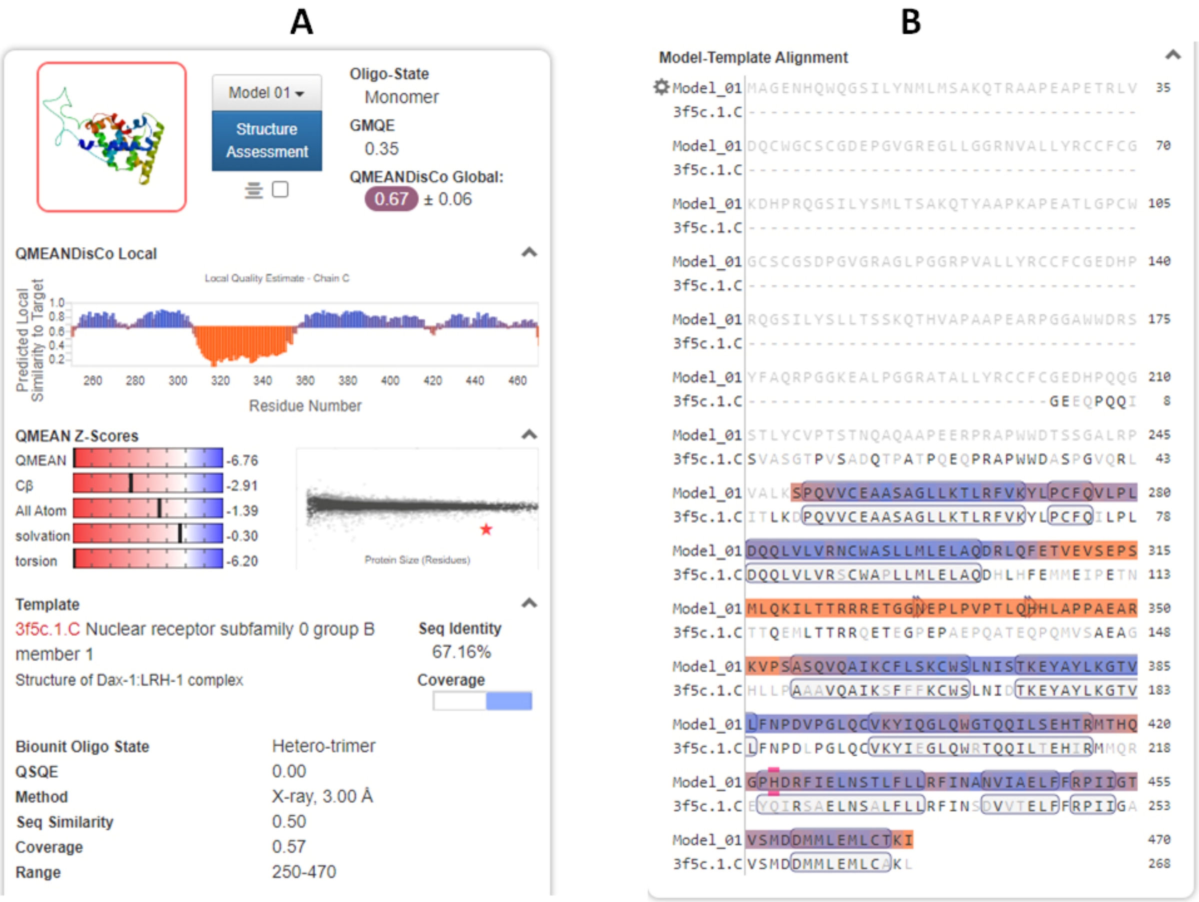

Figure S1. The figure shows the predicted DAX1 model in Figure A with 67.16 against the reference (PDB ID 3F5C) Mus Musculus while the sequence alignment of both proteins is depicted in Figure B.
